# Highly dynamic metabolic response of grapevine to water deficits reveals an adaptability to a wide range of climatic conditions

**DOI:** 10.1016/j.fochx.2026.104088

**Published:** 2026-06-17

**Authors:** Sébastien Nicolas, Benjamin Bois, Kévin Billet, Jenny Uhl, Olivier Mathieu, Anne-Lise Santoni, Roy Urvieta, Fernando Buscema, Manfred Stoll, Cornelis van Leeuwen, Philippe Schmitt-Kopplin, Régis D. Gougeon

**Affiliations:** a PAM, UMR 1517 INRAe, Université Bourgogne Europe, L'institut Agro, Institut Universitaire de la Vigne et du Vin – Jules Guyot, Rue Claude Ladrey, F-21000 Dijon, France; b Biogéosciences, UMR 6282 CNRS, université de Bourgogne Europe, 6 boulevard Gabriel, F-21000 Dijon, France; c Research Unit Analytical BioGeoChemistry, Helmholtz Zentrum München, Ingolstaedter Landstrasse 1, 85764 Neuherberg, Germany; d Catena Institute of Wine, Bodega Catena Zapata, Mendoza, Argentina; e Department of General and Organic Viticulture, Hochschule Geisenheim University, von-Lade-Straße 1, 65366 Geisenheim, Germany; f EGFV, Univ. Bordeaux, Bordeaux Sciences Agro, INRAE, ISVV, Chemin de Leysotte, F-33883 Villenave d'Ornon, France; g Analytische Lebensmittel Chemie, Technische Universität München, Maximus-von-Imhof-Forum 2, 85354 Freising, Germany

**Keywords:** Water status, Climate change, Metabolomics, ‘chardonnay’, ‘Pinot noir’, FT-ICR-MS

## Abstract

Changing climate conditions raise questions about the evolution of perennial grapevine varieties, which are grown in various places around the world, where complex interplay between environmental factors, genetics and viticultural practices can contribute to unexpected adaptability. Based on experimental measurements of water status (δ^13^C) and untargeted metabolomics of up to 256 grape juices from 13 wine regions in Europe and Argentina, over 3 successive vintages, we assessed the adaptability to climate change of ‘Pinot noir’ and ‘Chardonnay’ grapevines. Both varieties appeared to be able to withstand a wide range of water deficits, some of which may be associated with climatic conditions similar to a + 2 °C warming scenario in Burgundy. Multivariate models revealed hundreds of water-status-related mesocarp metabolites, with ‘Chardonnay’ showing a consistent dynamic response across the water status range, whereas many ‘Pinot noir’ markers faded under severe water deficit, suggesting this grape variety is less resilient to changing climatic conditions.

## Highlights

• Pinot Noir and Chardonnay face a wide range of water deficits, including severe conditions driven by global warming.
• Hundreds of mesocarp metabolites correlate with water deficit status, forming biochemical fingerprints of grape quality.
• Both varieties can grow under severe water stress, but Chardonnay is expected to fare better.
• Integrated metabolomics is able to capture relevant biochemical fingerprints from large sets of natural vineyard conditions.

## 1 Introduction

**Fig. 1:**
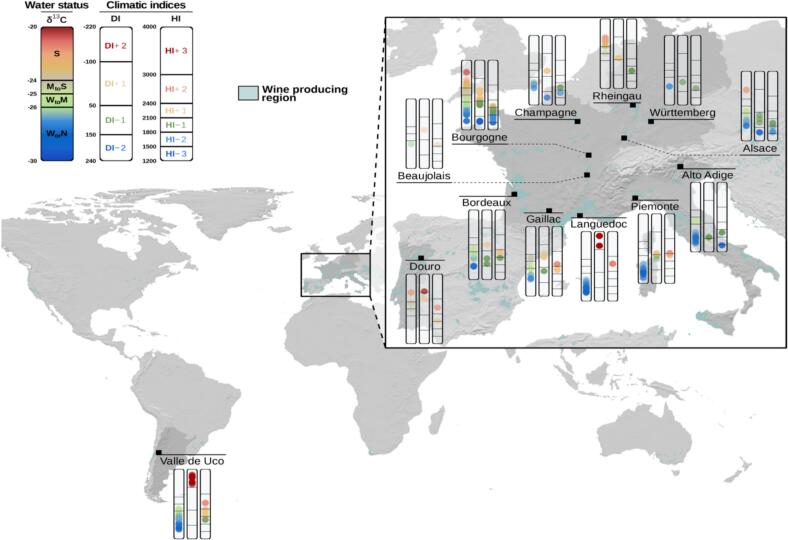
**δ**^**13**^**C and climatic indicators measured for the 13 wine producing regions.** Each colored plotted dot corresponds to one sampled site (vineyard). DI (mm): Dryness Index; HI (°C.days): Heliothermal Index. Climatic data have been retrieved from the Terra Climate database. δ^13^C classes are retrieved from Santesban's et al. classificaiton (W_or_N: Weak or Neal, W_to_M: Weak to Moderate, M_to_S: Moderate to Severe, S: Severe), while DI and HI classes are retrieved from Tonietto and Carbonneau's classification (DI-2: Humid, DI-1: Sub-humid, DI + 1: Moderately dry, DI + 2: Very dry; HI – 3: Very cool, HI – 2: Cool, HI – 1: Temperate, HI − +1: Temperate warm, HI − +2: Warm, HI − +3: Very warm).

**Fig. 2:**
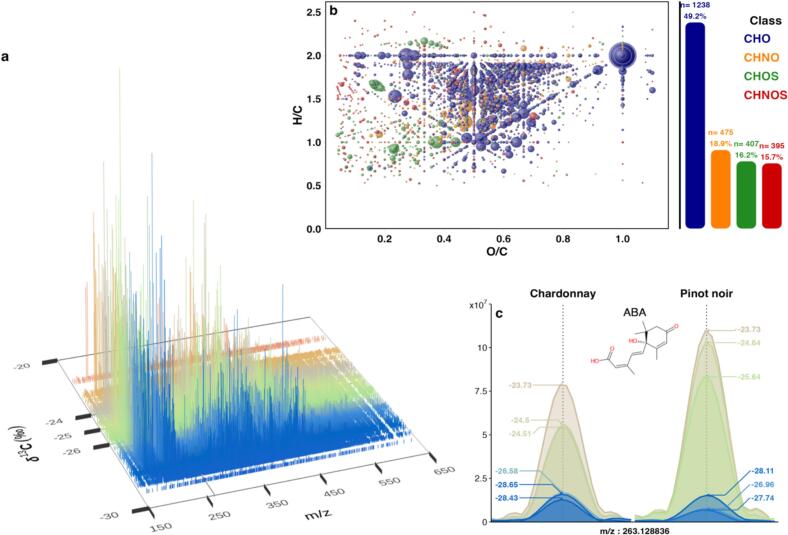
**Ultra-high resolution mass spectrometric analysis of grape juices.** a: 3-D representation of grape juices mass spectra sorted according to δ^13^C values in the 150–650 *m/z* range. The color code in a and c corresponds to the color gradient used in Fig. 1. b: 2-D van Krevelen diagram (H/C vs. O/C atomic ratios from assigned elemental formulas, bubble sizes correspond to relative mean peak intensities) and counts of individual elemental formulas within the four distinct Carbon (C), Hydrogen (H), Nitrogen (N), Oxygen (O) and Sulfur (S) chemical spaces (color code: CHO, blue; CHOS, green; CHNO, orange; CHNOS, red). c: focus on *m/z* 263.128836 measured intensity according to increasing δ^13^C values (from blue to green) of a subset of 6 ‘Chardonnay’ and 6 ‘Pinot noir’ samples. *m/z* 263.12884 is unambiguously assigned the [M-H]- ion with absolute mass formula [C_15_H_19_O_4_]-, possibly corresponding to abscisic acid (ABA).

**Fig. 3:**
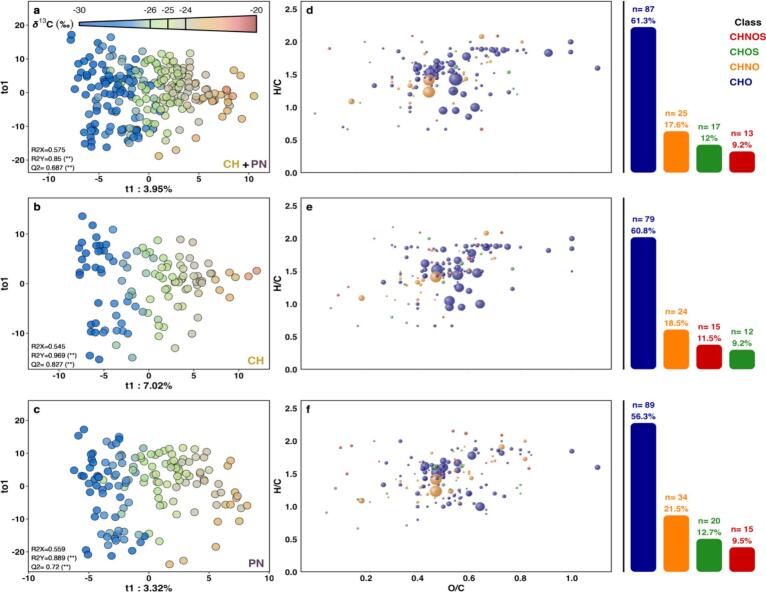
**O-PLS score plots colored according to δ**^**13**^**C values and associated, van Krevelen diagrams,** for ‘Chardonnay’ and ‘Pinot noir’ metabolomes pooled together (a), ‘Chardonnay’ alone (b), ‘Pinot noir’ alone (c). The color code in a to c corresponds to the color gradient used in Fig. 1 and ** corresponds to *p-values* < 0.01. d-f, van Krevelen diagrams and associated barplots representing VIPs >1.5 (mass peaks transformed into elemental formulas), whose relative intensity variation appeared either positively or negatively correlated with the increase of δ^13^C values. (color code: CHO, blue; CHOS, green; CHNO, orange; CHNOS, red).

**Fig. 4:**
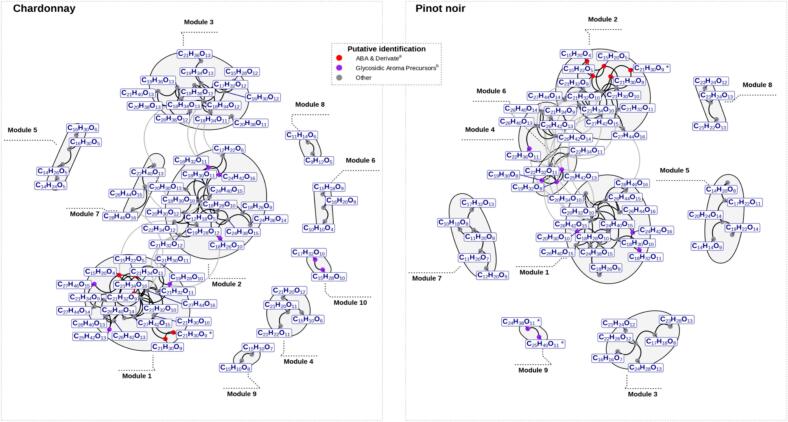
**Multilayer networks of VIPs restricted to the CHO class.** Multilayer network of VIPs computed for ‘Chardonnay’ (left) and ‘Pinot noir’ (right), coupling spearman correlation (|*ρ*| ≥ 0.7, q.value (FDR) < 10–5) and common metabolomic mass difference 41 on neutral elemental formulas. Chloride adducts were converted into their dedicated [M-H]- ions in silico and are indicated with *: ABA & derivate^a^: annotation using MetaCyc, PlantCyc and GrapeCyc database. Glycosidic Aroma Precursors^b^: annotation using compounds identified in (Caffrey et al., 2020; Cebrián-Tarancón et al., 2021; De Rosso et al., 2022; Hjelmeland et al., 2015; Wei et al., 2021).

**Fig. 5:**
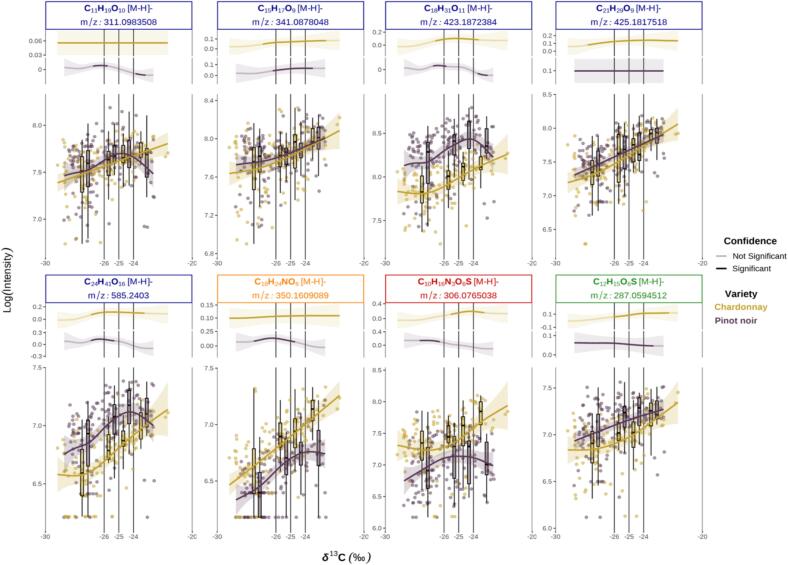
**Water status related dynamics of some VIP mass peak intensities, with assigned ion elemental compositions.** Each dot corresponds to the Log representation of the associated mass peak intensity for a given sample (location x vintage x cultivar). Curves are produced from general additive models (GAM) fitting each cultivar. The shaded areas along the lines correspond to the 95% confidence interval. Ion elemental formulas are colored according to their CHNOS class: CHO, blue; CHOS, green; CHNO, orange; CHNOS, red).

Water availability is of major importance in crop production. The combination of climate change and growing world population should lead to increased freshwater scarcity in many regions worldwide where agriculture is a major activity (Schewe et al., 2014), with highly uncertain economic outcomes (Dolan et al., 2021). At the dawn of the 21st century (1996–2005), 39 to 54% of global crop land was estimated to suffer water scarcity without possibilities to irrigate (Rosa et al., 2020). In these regions, the use of water-saving crops whose products have a high added value is relevant. This is the case of wine grape (*Vitis vinifera* L.) which is cultivated in a large range of water availability conditions, including dry areas (Puga et al., 2022). Water deficit, when not extreme, can increase wine quality (van Leeuwen et al., 2009). Thus, any losses in yield and hence turnover can very often be compensated for by the increased value of the resulting higher quality wine; this is particularly the case of red wine (Baciocco et al., 2014; van Leeuwen et al., 2009). Grapevine water status is thus often recognized as a key component of the so-called “Terroir” effect, a concept linking the taste of wine to its production location (White et al., 2009). (See Fig. 1, Fig. 2, Fig. 3, Fig. 4, Fig. 5.)

The impact of climate change on the evolution of grapevine physiology, in particular the effects of increasing temperatures and water deficits, have been extensively investigated over the past two decades (Charrier et al., 2018; Lovisolo et al., 2016; Marx et al., 2017; Pellegrino et al., 2005; Triolo et al., 2019; van Leeuwen & Darriet, 2016; Webb et al., 2012; Wolkovich et al., 2018). The results of most of these studies have pointed to the necessity for adaptation in many wine growing regions around the world due to the projected unsustainability of agriculture under climate change (Schultz & Jones, 2010). Key levers for adaptation include the selection of better-adapted varieties from either existing intraspecific diversity or new varieties obtained by breeding (Duchêne et al., 2010; Morales-Castilla et al., 2020; Sgubin et al., 2023; Wolkovich et al., 2018), and/or the implementation of alternative practices for water management such as irrigation (Gambetta et al., 2020; Miras-Avalos & Araujo, 2021).

However, these levers are considered as options of last resort in cool or temperate climate winegrowing regions bound by strict regulations such as the Loire valley (Neethling et al., 2017), Bourgogne or Champagne (Ollat et al., 2021) in France. A survey carried out in 2002–2003 revealed that 50% of winegrowers in France, Germany and Italy, would not consider changing the variety as a way of adapting to climate change (Battaglini et al., 2009). While many possibilities have been explored to adapt wine production to climate change(Naulleau et al., 2021), changing cultivars is indeed a risky choice for premium wine regions whose wine characteristics and reputations strongly rely on a single or a few varieties; this is because the combination of wine variety and place of origin strongly marks the wine sensory characteristics (Foroni et al., 2017; White et al., 2009). In this context, an increased understanding of the adaptability of long-lasting grapevine varieties with high added-value could contribute to developing innovative and knowledge-based winemaking practices; this would help winemaking regions maintain and enhance the natural resilience of their vines.

Questioning natural resilience for ‘Chardonnay’ and ‘Pinot noir’ grapevines is highly pertinent as these two varieties, emblematic of Bourgogne and Champagne, are grown over a wide range of latitudes and climates around the world (Carbonneau & Boidron, 2012; Gavrilescu & Bois, 2016; Shaw, 2012). If this climatic diversity can be used to characterize different wine growing regions and different terroirs (Jones, 2006; Puga et al., 2022; Tonietto & Carbonneau, 2004), it also results in diversity in the expression of their characters, which are all considered positive (Gavrilescu & Bois, 2016). Such characters are results of the interaction of the vine with its environment, leading to a modulation of the physiological response of the plant, which can be defined as phenotypic plasticity (Bradshaw, 2006; Sadras et al., 2009). Despite the debates that may exist around how to consider plasticity (Forsman, 2015; Gianoli & Valladares, 2012), the diversity of responses observed in the vine leaves little doubt when it comes to its aptitude for plasticity (Fernie & Tohge, 2013). The latter is therefore today a tool of choice to enable the assessment of the possible and ongoing impacts of climate change, through the measure of an in vivo physiological response of the vine to the complex mechanisms generated by exposure to biotic or abiotic stresses (Dal Santo et al., 2013).

Knowledge about the physiological traits and metabolic fingerprints of grapevine varieties, whether at berry or leaf level, has considerably increased because of various physiological and molecular-based studies. Mostly based on experiments carried out in controlled environments (Aru et al., 2022) or restricted to single vineyards (Savoi et al., 2020), these studies have produced important water-related findings, such as how the temporal aspect of water deficit impacts grapevines (Castellarin et al., 2007) and the numerous cultivar-dependent metabolic pathways potentially involved in grapevine resistance to water deficit (Cabral et al., 2022; Deluc et al., 2009; Mirás-Avalos & Intrigliolo, 2017; Pinasseau et al., 2017; Savoi et al., 2017; van Leeuwen et al., 2020). However, a specific variety can exhibit a wide range of physiological responses to water availability depending on all the environmental characteristics of the vineyard and their interactions (Hochberg et al., 2018), thus revealing the limits of the above-mentioned experimental setups when endeavoring to unravel the complex interplay between natural environmental factors, genetics and viticultural practices.

In the present study, we investigate how two emblematic grape cultivars ‘Chardonnay’ and ‘Pinot noir’ can thrive under a wide range of water statuses. Our study covered an international array of ‘Chardonnay’ and ‘Pinot noir’ vineyard environments whose current and future climatic conditions are representative of those of a region like Bourgogne. The juice obtained from the mesocarp of berries sampled at harvest over three successive vintages was measured for δ^13^C (‰)(Gaudillère et al., 2002; Santesteban et al., 2015), which is an indicator of vine water status; this indicator is also considered here as an integrated proxy for the physiological state of the vines. Untargeted metabolomics was also performed, combined with multivariate statistical analysis on these grape juices, revealing that the relative abundance of hundreds of metabolites is consistently correlated with water status. Ultra-high resolution mass spectrometry makes it possible to obtain instantaneous comprehensive metabolic fingerprints of grape juices, which are representative of many site-specific complex plant environment plant-environment interactions found under a wide range of vineyard conditions(Gougeon et al., 2009; Roullier-Gall et al., 2014; Schmitt-Kopplin et al., 2019).

## 2 Materials and methods

### 2.1 Sampling sites & methods

Ripe ‘Pinot noir’ and ‘Chardonnay’ grapes were harvested during three vintages (2019, 2020 and 2021) from a total of 80 vineyards in 13 wine producing regions of France, Italy, Germany, Portugal and Argentina. Each sample comprised randomly picked berries within the parcels, collected at harvest dates decided by each winemaker, to have juices genuinely representative of what is vinified. This may have introduced slight inter-regional variations in berry ripeness at harvest (e.g. earlier harvest in Champagne compared to Argentina), but this variability was considered intrinsic parameter of the sampling. The berries were frozen at −20 °C, to avoid any post-harvest variation when they had to be transported. Sample size allowing, pseudo-biological replicates were obtained by randomly choosing two pools of 100 berries; otherwise, only one pool of 100 berries was used per parcel. Each pool was then manually pressed, and the collected juice was immediately frozen before subsequent analyses (−20 °C).

### 2.2 Water status

The water status was evaluated by measuring δ^13^C (‰) following the protocol described by Gaudillère et al., 2002. Briefly, 5 μL of grape juice was pipetted into a tin capsule and placed in an oven at 40 °C for 12 h and analyzed in duplicate on a Vario Micro Cube elemental analyzer coupled in continuous flow mode to an isotope ratio mass spectrometer (IsoPrime, Elementar). USGS40 (IAEA, Vienna) was used as an internal standard (δ^13^C PDB = −26.392 ± 0.041‰). The δ^13^C values are reported in ‰, and each plot value corresponds to the mean of two technical replicates.

### 2.3 Ft-ICR-MS

Prior to the metabolomics analysis, grape juice samples were prepared by solid phase extraction (Bond Elut C18 cartridge, 100 mg, 1 mL, 120 μm, Agilent). After thawing, the samples were vortexed and centrifuged (10 min, 10000 rpm, 4 °C), and a pH of 2 was obtained using Formic Acid. The cartridges were conditioned with 2 mL methanol (Honeywell), followed by 1 mL acidified ultra-pure water (18.2 MΩ, Millipore, formic acid). Samples were then passed through each cartridge using CHROMABOND SPE vacuum manifold and eluted with 1 mL of methanol and stored in vials at −20 °C.

For the Fourier Transform Ion Cyclotron Resonance Mass Spectrometer (FT-ICR-MS) analysis, samples were diluted (1/20) in methanol. Mass spectra were acquired in negative ionization mode by a 12 T FT-ICR-MS, Bruker SolariX ultra high-resolution mass spectrometer (Bruker Daltonics, Bremen, Germany), with a flow rate of 120 μL.h − 1. Spectra were acquired with the accumulation of 400 scans over a 92–1000 *m/z* mass range. Raw Spectra were calibrated using Compass DataAnalysis 4.2 (Bruker Daltonics, Bremen, Germany) and peaks with a signal-to-noise ratio (S/N) of at least 3 were considered. A matrix was then obtained by aligning all spectra within a margin of error of 0.5 ppm and molecular formulae were assigned using an in-house developed software tool (Tziotis et al., 2011).

### 2.4 Statistical analyses

All the statistical analyses and posts treatments were performed within the R environment 61 (v 4.3.0). Variance partitioning was performed using Vegan package (Oksanen et al., 2022). In our study, 3 distinct metabolomic datasets were considered, consisting of either all samples (i.e., the pool: ‘Chardonnay’ and ‘Pinot noir’) or variety specific (i.e., ‘Chardonnay’ and ‘Pinot noir’ respectively) datasets. Prior to any statistical analyses, the batch effect (2 Batches) was corrected using the DBnorm package with ber method (Bararpour et al., 2021; Giordan, 2014). A data sanity check was also performed, resulting in two samples being removed (Thevenot et al., 2015). Before each statistical treatment, zero values were replaced with 2/3 of the minimum value of a given feature. Datasets were then Log10 transformed, pareto scaled and filtered to keep *m/z* present in more than 33% of the samples.

Supervised multivariate statistics were performed using ropls package using O-PLS (with δ^13^C as the response) or O-PLS-DA (with variety as the response)(A. Thevenot et al., 2015). To assess the significance of the model permutation tests (n_perm_ = 500) were applied. The differences in intensity of the varieties (fold change) were tested using Student *t*-test. For the multilayer network, Spearman correlation was used as a measure of similarity and only |*ρ*| ≥ 0.7 were kept, and the mass differences were restricted to the list provided in the literature (Breitling et al., 2006). For both the Student t-test and Spearman correlation, the p.values were corrected (p.adjust) using the False Discovery Rate method (FDR) and *p.adjust* < 0.05 were considered significant. For the Generalized Additive Models (GAM), the mgcv package was used (Wood, 2011), and marginal derivatives were used to infer model significance along the δ^13^C gradient (Makowski et al., 2020), with REML smoothing parameter estimation method.

Finally, the measured *m/z* were annotated using the MetaCyc (V26.0), Plant Metabolic Network (PMN, V15.0) and GrapeCyc (V9.0.1) databases (Caspi et al., 2020; Hawkins et al., 2021), along with the MetaboAnnotation package (Rainer et al., 2022).

## 3 Results

### 3.1 A wide range of grapevine water status conditions exhibited by wine producing areas

Fig. 1 shows dot plots of δ^13^C values (256 samples, nCH =112; nPN = 144, Table S1–2), along with corresponding heliothermal and dryness indexes (Fig. S1–3) for the 13 different ‘Chardonnay’ and ‘Pinot noir’ wine-producing regions from 5 different countries and 3 consecutive vintages (2019, 2020 and 2021), and for a total of 80 parcels. With δ^13^C values ranging from −29.2‰ (Piedmont, IT, 2020) to −21.6‰ (Burgundy, FR, 2020), the ‘Chardonnay’ variety exhibited a slightly wider water status range than ‘Pinot noir’ (−28.8‰ for Alto Adige, IT, 2019 to −22.6‰ for Rheingau, DE, 2019). This water status variability appears to be mostly linked to area of production (60.5%) and vintage (18.3%), while the effect of variety was almost non-existent (Fig. S4). Based on the relationship between δ^13^C values and water deficit described by Santesteban et al., 2015, our results indicate that for the three vintages in question, a cool region like Burgundy (HI-3 to HI + 1 and DI-2 to DI + 1) can undergo very different grapevine water deficits: from Nil or Weak (N_or_W, 2021) to Severe (S, 2020), with the 2019 vintage being intermediately associated with Weak to Moderate (W_to_M) and Moderate to Severe (M_to_S) deficits (Fig. 1). Interestingly however for Burgundy, if the 2020 vintage exhibited the highest water deficit (average value of −24.5‰), the 2019 vintage appeared to be the driest (DI + 1), and this trend was also observed in Bordeaux, but not in Champagne (Fig. S5). By contrast, and because of the implementation of complementary irrigation due to very low rainfall, the very dry highly elevated region of Valle de Uco in Argentina (DI + 2 and HI-1 to HI + 2) did experience only N_or_W to W_to_M water deficits, (Table S1). Overall, not only is the explored spatial diversity here associated with almost the whole water status range, but the δ^13^C values provide snapshots of the real grapevine responses to complex plant-soil-climate interactions, where it appears to better explain the berry composition than does temperature, as well as being associated with three vintages characterized by very different climatic conditions (Fig. S6–7).

### 3.2 Revealing grape juice metabolomes to be driven by water status

The non-targeted metabolomics analysis by ultra-high resolution mass spectrometry (mostly distributed within the 100–650 *m/z* range, 98%) of the 256 grape juices resulted in the detection of up to 7530 distinct *m/z* peaks in at least 2 samples (Fig. 2a); of these 6245 could be assigned unique CHNOS-containing elemental formulas likely representing chemical fingerprints of several tens of thousands of compounds(Gougeon et al., 2009).

‘Pinot noir’ juices were characterized by up to 6325 *m/z* values, whereas ‘Chardonnay’ juices were characterized by 4845 *m/z* values, both with a significantly high CHO abundance (35.61% and 38.49% respectively). A supervised (O-PLS-DA) analysis of the entire metabolomics dataset (Fig. S8) clearly discriminated the two varieties, with 167 Variable Importance in Projection (VIP) > 1.5 comprising largely CHO elemental formulas (72.9%); of the 167 VIPs, 125 versus 41 biomarkers identify ‘Pinot noir’ and ‘Chardonnay’ respectively. Besides the various chemical families revealed by the two-dimensional van Krevelen diagram (Fig. 2b), including fatty acids, peptides, carbohydrates, organic acids and derivatives, the juice fingerprints were characterized by extensive series of glycosylation homologues (Gougeon et al., 2009).

The O-PLS multivariate analysis of the metabolomics and δ^13^C datasets revealed a significant (*p* < 0.01) and clear discrimination of the juice metabolomes depending on the δ^13^C values, both for ‘Chardonnay’ and ‘Pinot noir’ alone, as well as for the two cultivars pooled together (Fig. 3). The total of 307 CHNOS mass values with VIP > 1.5 can explain the relationship between.

the juice metabolomes and vine water status (nAll = 142, nCH = 130, nPN = 158, Fig. 3 a-c), most of them positively correlating with the increase in δ^13^C values (Fig. 5 and Fig. S9). Most interestingly, the O-PLS model with pooled cultivars successfully identified 65.6% and 59.8% of the VIPs revealed by the ‘Chardonnay’ model or ‘Pinot noir’ model alone respectively. Altogether, these VIPs mostly corresponded to CHO-containing compounds (56.3%–61.3%), followed by CHNO (17.6% -21.5%), CHOS (9.2%–12.7%) and CHNOS (9.2%–11.5%) (Fig. 3 d-f).

However, each cultivar exhibited specific fingerprints, with 63.1% and 68.8% of VIPs being detected only in ‘Chardonnay’ and ‘Pinot noir’ grape juices respectively. Among these “variety specific” elemental compositions (*n* = 231), 141 CHNOS elemental composition were found significantly more present (*p* < 0.05, Fig. S10) in either ‘Chardonnay’ (37.6%) or ‘Pinot noir’ (62.4%). By searching databases, we found several putative annotations consistent with compounds previously described for their response to water status (Fig. S9). For instance, the peak at mass 263.128836 that can unambiguously be assigned to the [M-H]- ion with absolute mass formula [C_15_H_19_O_4_]- and matching abscisic acid (ABA) is an example of the numerous mass peaks whose relative intensity increased consistently with δ^13^C value (i.e., the relative intensity was higher for less negative δ^13^C values) for both cultivars (Fig. 2). Phaseic acid (PA), ABA-glucose ester (ABA-GE), Catechin, Quercetin-3-*O*-Glucoside, Kampferol-3-*O*-glucoside are other examples of database hits (i.e. matching exact absolute mass formulas found in variety specific fingerprints), possibly corresponding to compounds described in the literature as being related to vine water status (Deluc et al., 2009; Savoi et al., 2016; Toups et al., 2022) (Table S3).

### 3.3 The diversity of plant-growth regulators and sensory-impact metabolites correlated with vine water status

In both cultivars, the co-occurrence networks built on the correlations between the VIPs mass peak values revealed several densely correlated sub-networks of elemental formulas within the CHO chemical space, and to a lesser extent within the other CHNO, CHOS and CHNOS chemical spaces (Fig. S11 and S12). All these networks connected the mass peaks whose intensity variation was correlated with the water status of the grapevine. Fig. 4 shows a conjugation of co-occurrences with exact mass differences within the most abundant CHO chemical space. It reveals patterns of elemental formulas which can be assigned to ABA and its derivatives, exhibiting connections, such as hydration [+H_2_O], hydrogenation [+H_2_] and glycosylation [+C_6_H_10_O_5_], as observed for the putative PA [C_15_H_20_O_5_] and ABA [C_15_H_20_O_4_] in both cultivars. Similarly, several other elemental formulas can tentatively be assigned to glycosidic aroma precursors recently observed in grapes (Caffrey et al., 2020; Cebrián-Tarancón et al., 2021; De Rosso et al., 2021; Hjelmeland et al., 2015; Wei et al., 2021), including monoterpenoid glycosides [C_21_H_34_O_11_] or [C_27_H_46_O_15_], norisoprenoid hexose [C_25_H_42_O_13_] and aliphatic alcohol glycosides [C_17_H_32_O_10_] or [C_18_H_34_O_10_]. Interestingly, the two latter elemental formulas, which are connected by a [CH_2_] difference, possibly refer to a glycosidic precursor and its methylated form. Similarly, our tentative assignments are reinforced by the observation of the [C_24_H_42_O_16_] and [C_18_H_32_O_11_] elemental formulas, which are connected by a [C_6_H_10_O_5_] difference, and which can be assigned to a trisaccharide precursor (hexose-hexose-hexose-C6) and a disaccharide precursor (hexose-hexose dehydro-C6) respectively (Caffrey et al., 2020). Finally, while the CHNO chemical space also shows dense interconnections between peptide-like compounds, it is worth mentioning that in the CHNOS chemical space of the ‘Chardonnay’ samples, the [C_10_H_17_N_3_O_6_S] elemental formula likely assignable to glutathione also exhibited various connections; this indicates the dependence of sulfur-containing peptide concentrations to water status. The richness of our metabolomics data allowed us to identify hydroxylation as the most abundant mass differences of the ‘Chardonnay’ water status related VIPs, whereas it was hydrogenation/dehydrogenation for ‘Pinot noir’ (Fig. S11 and S12). Hence, metabolomic fingerprints linked to vine water status are variety specific.

Within the explored water status range, the two grapevine cultivars exhibited different metabolic responses to water deficit. As shown in Fig. 5 (see Fig. S13 for complete overview), the actual dependence of the detected metabolites to water status can exhibit distinct variation dynamics (variation of the corresponding mass peak intensity with an increase in water deficit characterized by less negative δ^13^C values) over the entire range of δ^13^C values. Within a 95% confidence interval, positive trends (relative peak intensity increase) were generally comparable for the two cultivars over the entire range of δ^13^C values, and within all CHONS classes, as exemplified by the assigned [C_15_H_17_O_9_]-, [C_21_H_29_O_9_]-, or [C_18_H_24_NO_6_]- ion elemental formulas (Fig. 5), although the variation was not always significant over the entire water status range. The two former ion formulas may both possibly correspond to glycosides (caffeic acid and ABA respectively), whereas the latter may correspond to an amino acid derivative (Table S3). By contrast, for ‘Pinot noir’, several non-monotonic peak variations appeared to significantly diverge above −24‰; i.e., in the case of severe water deficits (Fig. S13). The assigned [C_11_H_19_O_10_]- and [C_18_H_31_O_11_]- ion elemental formulas (Fig. 5) are examples of this behavior: the relative concentration of metabolites increases with an increase in water deficit throughout the entire δ^13^C range for ‘Chardonnay’, whereas they start to decrease above *ca* − 25 / -24‰ for ‘Pinot noir’. Putative annotations for these ion formulas would be glycosides, with an arabinofuranosylglucoside in the case of the former, and a disaccharide aroma precursor (hexose-hexose dehydro-C6), which have been shown to be involved in grape aroma glycosylation (Gunata et al., 1990). It should be noted that the former [C_11_H_19_O_10_]- ion is a VIP for ‘Chardonnay’ only, and its observed dynamics (non-monotonic with a switch-off above −24‰) could be related to the lower t1 component of the O-PLS model for pooled and ‘Pinot noir’ samples (Fig. 3). Interestingly, the [C_10_H_16_N_3_O_6_S]- ion, likely corresponding to glutathion, exhibited a significant increase over −26‰ for ‘Chardonnay’. It must be noted that many constant or negative trends (relative peak intensity stability or decrease) were also observed for the two cultivars, thus witnessing the complex interplays involved in the metabolic response to water deficit (Fig. S13). The [C_15_H_11_O_6_]- ion elemental formula, putatively assigned to the eriodictyol flavonol, previously identified in ‘Pinot noir’ hairy root culture extract (Tisserant et al., 2016), is an example of mass whose intensity is constant for δ^13^C values below −25‰ for the two cultivars, but which significantly decreases above for ‘Pinot noir’ only (Fig. S13).

## 4 Discussion

Investigating the potential for growing grapevines in contemporary and possibly new wine producing regions is of major importance for an industry of high economic impact in many countries around the world (Bisson et al., 2002). A lot of studies have modeled the evolution of climatic parameters within the framework of climate change scenarios; these studies generally conclude that in wine growing regions with high added-value, such as Bourgogne, adaptation will rely on either the planting of new varieties or the development of innovative management strategies that involve, for example, the implementation of supplementary irrigation or reducing plant spacing (van Leeuwen et al., 2024). Furthermore, various experiments have been carried out in controlled conditions to extensively study the impact of heat or water stress on grapevine physiology and/or metabolism (Cabral et al., 2022; Hewitt et al., 2023; Savoi et al., 2017, 2020). Whether through modeling or experimentation, such studies contribute to a better understanding of the adaptability of grapevines; however, the model-based studies do not fully consider the complexity of plant-environment interactions, and the experiment-based studies have not been able to fully address the variability of environmental conditions across vineyards. Here, we determined the correlations between observed δ^13^C values - considered as an integrative proxy of vine water status - and metabolic responses in ‘Chardonnay’ and ‘Pinot noir’ grape juices. We carried out ultra-high-resolution mass-spectrometry analyses on the same grape juices made from berries sampled from vineyards in 13 different locations in Europe and Argentina over three consecutive vintages. A combination of metabolomics and bioinformatics was applied to obtain snapshots of metabolic fingerprints of grape juices, which are considered to comprehensively integrate all plant-environment interactions associated with multiple vineyard conditions in a system-biology approach (Nicholson & Lindon, 2008). In our experimental setup, variables representing environmental conditions (i.e., climatic parameters, geographical variables, such as vineyard elevation and soil characteristics, and biological variables, such as clones, rootstocks and the vine age) are thus integrated into the metabolic responses represented by biological indicator of the environmentally related water status of the vine. It was thereby possible to relate specific molecular fingerprints and their correlated dynamics to environmental conditions and vineyard management practices, such as irrigation.

Water deficit is considered a key driver of wine quality and terroir expression. Our results show that over three consecutive vintages (2019–2021) the two emblematic and genetically related cultivars (Bowers et al., 1999) were exposed to a wide water status range (Fig. 1), from Nil or Weak to Severe according to Santesteban's classification (Santesteban et al., 2015). Across all regions, the ‘Chardonnay’ cultivar underwent the widest range of water deficits, which is consistent with its cultivation in a wide range of climatic conditions (Gavrilescu & Bois, 2016). Our results also show that the driest regions, such as the Languedoc region in France and the Valle de Uco in Argentina, are not those in which the vines are exposed to the highest water deficits due to the application of irrigation. By contrast, dry-farmed vineyards, such as the vineyards in Bourgogne (which provided the highest number of samples), showed the full range of grapevine water deficits, from Nil or Weak in 2021 to Severe in 2020 (Fig. 1). Interestingly, if the 2020 vintage in Bourgogne appeared to be among the warmest since the 1970s, it appeared to be less dry than the 2019 vintage (Fig. S5). The 2020 harvest of the ‘Chardonnay’ grapes started near Beaune on August 18th (September 4th in 2019), an unprecedented early harvest according to available records for this region dating back to the 14th century (Labbé et al., 2019) (Table S4). Indeed, 2020 climate conditions are similar to a projected average vintage in a + 2 °C (relative to the pre-industrial era) warming scenario for Bourgogne, as well as for Bordeaux, Champagne and Valle de Uco in Argentina (Table S5). Therefore, with the Bourgogne case, and to a lesser extent the Bordeaux case (fewer data points), our results confirm that the water status, expressed as δ^13^C values, better integrated the genuine biological state of the vine for these two vintages, than would do monthly recorded climatic index such as DI.

Using a non-targeted approach combined with the unique resolving power of FT-ICR-MS, a remarkable diversity of secondary metabolites was detected in the grape juices, with unique CHNOS-based elemental formulas being assigned to up to 6993 *m/z* peaks. Mostly composed of metabolites from the CHO chemical space (Fig. 2), the complex molecular compositions of the ripe grape juices were considered to have fingerprints specific to water status and to distinct climatic conditions. δ^13^C values appeared to better explain our metabolomics dataset than climatic indices, such as HI or DI (Fig. S6). The results obtained from the O-PLS-based models indicate that up to 256 *m/z* peaks, and thus likely over a thousand metabolites, correlate with water status (Fig. 3, 4); these metabolites are possibly comprising plant-growth hormones, such as ABA and derivatives, glycosidic aroma precursors, peptides and phenolics, which can all potentially be used as markers of the adaptability of ‘Chardonnay’ and ‘Pinot noir’ grapevines to abiotic stresses, such as water deficit. In agreement with literature (Caffrey et al., 2020; Cebrián-Tarancón et al., 2021; De Rosso et al., 2022; Toups et al., 2022; Wei et al., 2021), we provide further evidence that the concentration of many sensory-related metabolites in grape juice is positively modulated by water deficit. It should be noted that hits were obtained for only 20.7% of the assigned elemental formulas of interest in the available databases, thus indicating that most of these markers are still unknown (Fig. S9).

In the case of ‘Chardonnay’, the positive impact of water deficit on glutathion and peptide derivatives provides new evidence of the link between water status and climatic conditions in general and the oxidative stability of dry white wines (Romanet et al., 2023). The increase in peak intensity assigned to glutathion with δ^13^C values above −26‰ (Fig. 5), along with connected VIPs within the CHNOS chemical space (Fig. S10), indicates that current and future climatic conditions contribute to the increased production of the native grape antioxydant metabolome regarding dry ‘Chardonnay’ wines (Romanet et al., 2020). As regards glutathion, various metabolites from the different CHNOS chemical spaces exhibited similar concentration dynamics (mostly increasing trends) in the two cultivars for Nil to Moderate stress; however, some of them differed (decreasing trend) in ‘Pinot noir’ when the water deficit was high (δ^13^C > −24‰, Fig. 5), confirming that this cultivar may have lower plasticity to water deficits. Such sensitivity of ‘Pinot noir’ to environmental conditions has previously been reported when comparing elemental and metabolite compositions with sensory profiles in western USA and Europe (Cantu et al., 2021; Castagna et al., 2017; Grainger et al., 2021; Nicholas et al., 2011). By contrast, subject to the explored water status range, the ‘Chardonnay’ cultivar did not seem to reach a tipping point, and the response dynamics of the metabolites was linear, suggesting a potentially higher adaptability of this variety to its changing environment. The fact that a higher number of metabolites in the ‘Chardonnay’ grape juices responded linearly to the water deficit may also be the reason why the O-PLS models (Fig. 3) provided a better variance explanation for this cultivar (7.05%) than for ‘Pinot noir’ (3.29%)(Wold et al., 2001). In addition, the supervised models, which are based on different water status levels (Fig. S14–16), also showed a possible response of polyphenolic compounds such as anthocyanins in ‘Pinot noir’ (Gougeon et al., 2009). A decrease in berry size due to water stress may also contribute to this relative increase in peak intensity but is not sufficient to explain the relative decreases also observed for many metabolites (Fig. S13). Our results are consistent with those of previous studies in which targeted analyses of volatile organic compounds, tannins, anthocyanins and metabolic pathways helped identify cultivar-specific compositions in controlled conditions (Deluc et al., 2009; Savoi et al., 2020). Nevertheless, here, it is the first time that consistent correlations have been found under a wide range of water conditions and in real production contexts.

Despite having gathered a remarkably large dataset of ‘Chardonnay’ and ‘Pinot noir’ vineyard characteristics and climate conditions, some comments can be made regarding the limits of our results. In Argentina, the severe water deficit was compensated for by irrigation, thus regions such as Bourgogne, Bordeaux, Champagne and Valle de Uco could all be described as having had moderately dry climate conditions, although Bourgogne, Bordeaux and Champagne were previously classified as sub-humid regions. Therefore, our dataset lacked grape juices made from berries from severely stressed vines, highlighting that natural experimental conditions can put unwanted limitations on an integrated approach. All the same, they reveal the reality of environmental conditions, which can significantly deviate from model projections based on the sole climatic indices. Furthermore, δ^13^C values can only be considered as indicators of vine water status during sugar accumulation in berries (Gaudillère et al., 2002); therefore, once again, natural experimental conditions will be defined by the physiology of the berry at harvest, which is highly variable. Nevertheless, even without a controlled water deficit range, but with a high natural variability within the sampling site characterized by different plant materials (rootstocks, scions and clones), grapevine ages, vineyard training systems, ripening speeds and durations, we reveal for the first time that consistent metabolic responses over a wide water deficit range can be observed; δ^13^C provided a better explanation of the variance of metabolomics data than widely-used climatic indices (Fig. S5). This result was obtained via the unique resolving power and extensive dynamic molecular range of the non-targeted metabolomics revealed by ultra-high-resolution mass spectrometry (Schmitt-Kopplin et al., 2019).

Therefore, our study shows that alongside existing model-based projections combined analytical approaches based on real vineyard conditions contribute to improving knowledge of the grapevine's adaptability to climate change. Future additional data acquisition is required to consolidate this model.

## 5 Conclusions

By integrating δ^13^C-based water status measurements of vines, untargeted ultra-high-resolution metabolomics of related grape juices and advanced multivariate statistics across 13 wine-producing regions and three consecutive vintages, this study reveals that both ‘Chardonnay’ and ‘Pinot noir’ grapevines possess a remarkable capacity to adapt to a wide range of water deficit conditions, including those associated with a + 2 °C warming scenario for Burgundy, through highly dynamic metabolic responses. Hundreds of metabolites spanning potential plant-growth regulators, glycosidic aroma precursors, phenolics, and antioxidant compounds were shown to be consistently modulated by the vine water status, demonstrating that δ^13^C is a more integrative and biologically informative proxy than conventional climatic indices alone. These results show that the two cultivars diverge under severe water deficit: while ‘Chardonnay’ sustains a mostly linear metabolic response throughout the full stress range, suggesting robust phenotypic plasticity, ‘Pinot noir’ chemical markers progressively fade beyond −24‰, indicating a potential physiological tipping point and greater vulnerability to extreme drought.

Beyond their physiological significance, these findings have direct implications for winemaking: the water-status-dependent modulation of a large set of grape juice metabolites, some of which putatively associated with aroma precursors and antioxidant metabolites, shows that increasing abiotic water stress resulting from changing climate is susceptible to gradually modify the wine organoleptic properties. For ‘Chardonnay’ wines, and within the water status range explored here, the sensory potential would evolve in such a way that aroma traits could progressively be perceived as remnants of known and generally shared properties, because of gradual monotonic modification (gain or loss) of grape juice aroma precursors. In contrast, the sensory potential of ‘Pinot noir’ wines would evolve in the same way only over a narrower range of water status for the vine. Our results suggest that above a certain severity of water deficit, ‘Pinot noir’ wines would exhibit more contrasted or unknown properties because of a sudden switching-off of metabolic pathways during ripening. Overall, this study shows that extremely rich metabolic signatures can now serve as decision-making tools for adaptive viticultural and oenological strategies aimed at controlling the quality and typicity of wines in a changing climate.

## CRediT authorship contribution statement

**Sébastien Nicolas:** Writing – original draft, Visualization, Validation, Software, Resources, Methodology, Investigation, Formal analysis, Data curation, Conceptualization. **Benjamin Bois:** Writing – original draft, Visualization, Validation, Supervision, Software, Resources, Project administration, Methodology, Investigation, Funding acquisition, Formal analysis, Data curation, Conceptualization. **Kévin Billet:** Writing – original draft, Investigation, Formal analysis, Data curation. **Jenny Uhl:** Writing – original draft, Software, Resources, Data curation. **Olivier Mathieu:** Software, Resources, Formal analysis. **Anne-Lise Santoni:** Software, Resources, Formal analysis. **Roy Urvieta:** Writing – original draft, Resources, Investigation, Data curation. **Fernando Buscema:** Writing – original draft, Resources. **Manfred Stoll:** Writing – original draft, Resources. **Cornelis van Leeuwen:** Writing – original draft, Validation, Resources. **Philippe Schmitt-Kopplin:** Writing – original draft, Visualization, Validation, Supervision, Software, Resources, Methodology, Investigation, Funding acquisition, Formal analysis, Data curation, Conceptualization. **Régis D. Gougeon:** Writing – original draft, Visualization, Validation, Supervision, Software, Resources, Project administration, Methodology, Investigation, Funding acquisition, Formal analysis, Data curation, Conceptualization.

## Funding

This work was funded by the Bureau Interprofessionnel des Vins de Bourgogne (BIVB, CLIMCHANGE project), the Comité Champagne (CLIMCHANGE project), the Catena Institute (CLIMCHANGE project), and the Conseil Régional de Bourgogne - Franche-Comté and the European Union through the PO FEDER-FSE Bourgogne 2014/2020 (METABOLOM project, grant BG0022832).

## Declaration of competing interest

The authors declare that they have no known competing financial interests or personal relationships that could have appeared to influence the work reported in this paper.

## Data Availability

Data will be made available on request. cloud UBESupplementary materials (Reference data)
