# Supplementary material for: Highly dynamic metabolic response of grapevine to water deficits reveals an adaptability to a wide range of climatic conditions
Source: Food Chem X. 2026 Jun 17;37:104088. doi: 10.1016/j.fochx.2026.104088 (PMC13311793; doi:10.1016/j.fochx.2026.104088)
Supplement: Supplementary file 1 — Supplementary material 1 [file mmc1.docx]

Supplementary Materials for

**Highly dynamic metabolic response of grapevine to water deficits reveals an adaptability to a wide range of climatic conditions**

Sébastien Nicolas, Benjamin Bois, Kévin Billet, Jenny Uhl, Olivier Mathieu, Anne-Lise Santoni, Roy Urvieta, Fernando Buscema, Manfred Stoll, Cornelis van Leeuwen, Philippe Schmitt-Kopplin, Régis D. Gougeon

Fig. S1. Boxplot representations of all δ^13^C measurements for all locations and vintages. Each dot corresponds to one sample (one site and one vintage). The upper part of the figure shows the density curve of the δ^13^C distribution for Chardonnay (yellow), Pinot noir (dark red) and all the samples independently of the variety (grey). Horizontal lines inside the distributions indicate from left to right, quartile 1, median and quartile 3. The lower part of the figure presents the water deficit classes based on the threshold described bySantesteban et al., 2015, and the color gradient associated with the δ^13^C values.


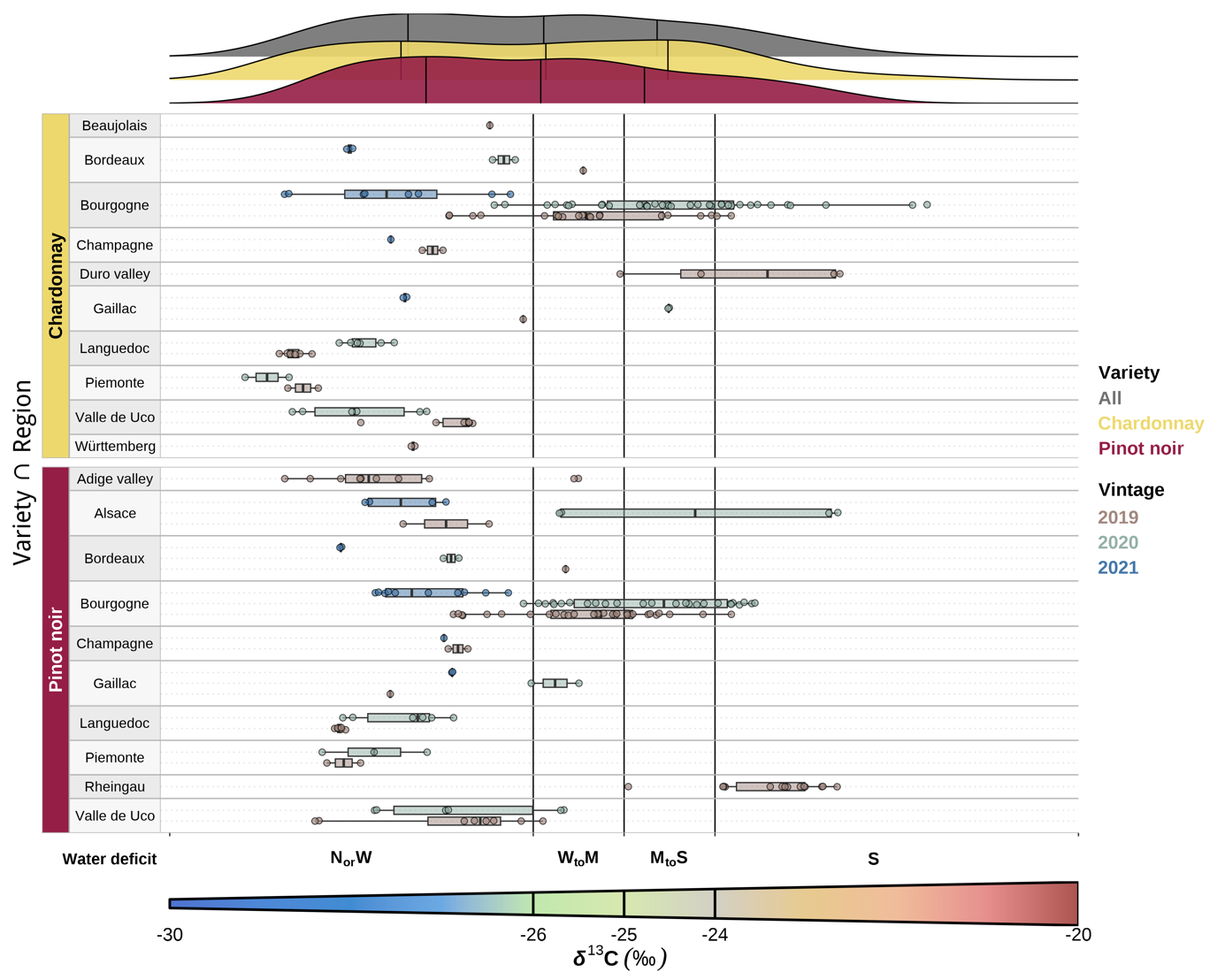


Fig. S2. Boxplot representations of the Dryness Index (DI) for all locations and vintages. Each dot corresponds to one sample (one site and one vintage). The upper part of the figure shows the density curve of the DI (Riou, 1994), calculated for Chardonnay (yellow), Pinot noir (dark red) and all the samples independently of the variety (grey). Horizontal lines inside the distributions indicate from left to right, quartile 1, median and quartile 3. The lower part of the figure presents the DI classes based on Tonietto & Carbonneau, 2004, with the following classification for grape-growing regions: DI-2: Humid; DI-1: Sub-humid; DI+1: Moderately dry; DI+2: Very dry.


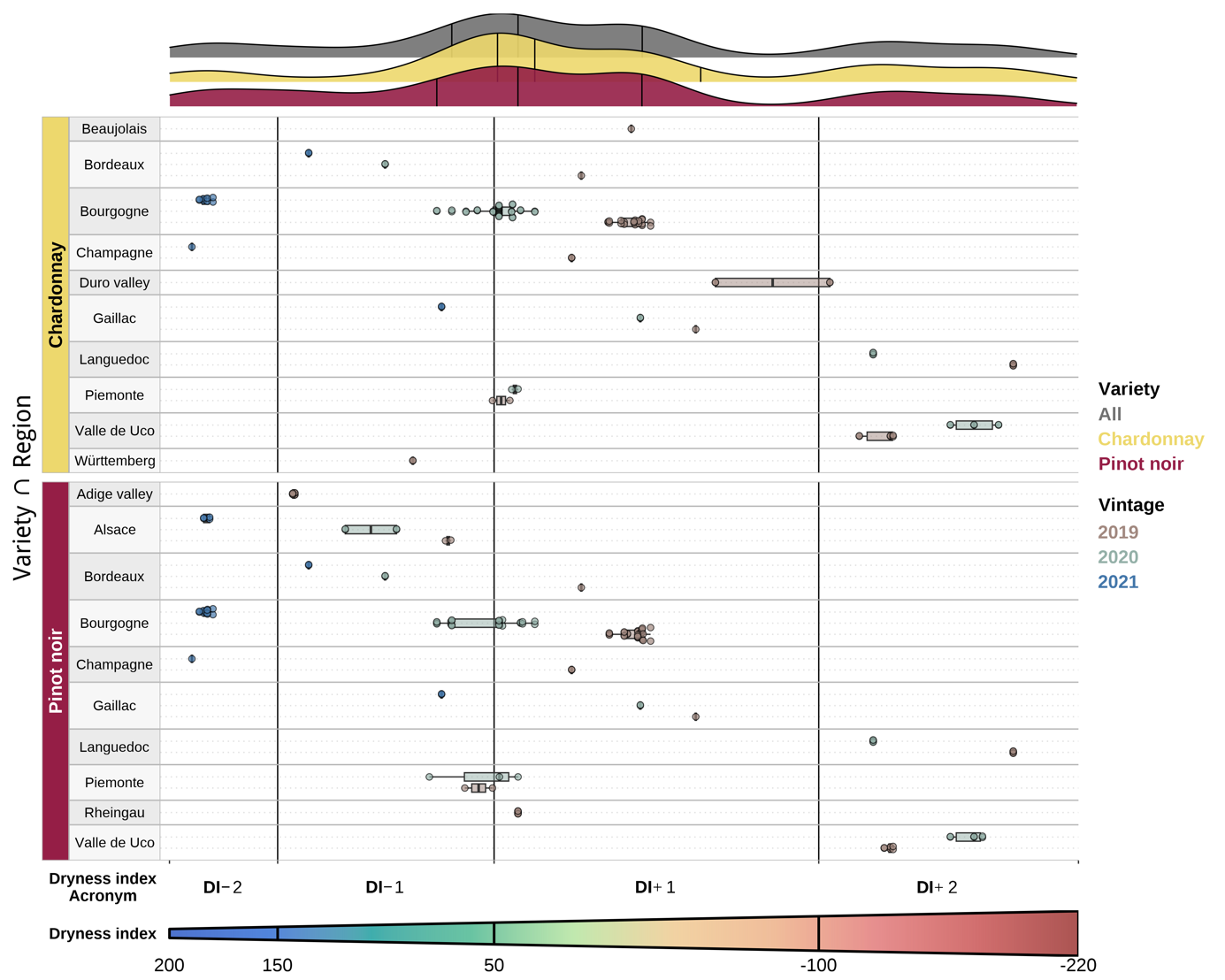


**Fig**. **S3**. **Boxplot representations of the Huglin Index (HI) for all locations and vintages.** Each dot corresponds to one sample (one site and one vintage). The upper part of the figure shows the density curve of the HI (Huglin, 1978), calculated for Chardonnay (yellow), Pinot noir (dark red) and all the samples independently of the variety (grey). Horizontal lines inside the distributions indicate from left to right, quartile 1, median and quartile 3. The lower part of the figure presents the HI classes based on Tonietto & Carbonneau, 2004, with the following classification for grape-growing regions: HI-3: Very cool; HI-2: Cool; HI-1: Temperate, HI+1: Temperate warm, HI+2: Warm, HI+3: Very warm.


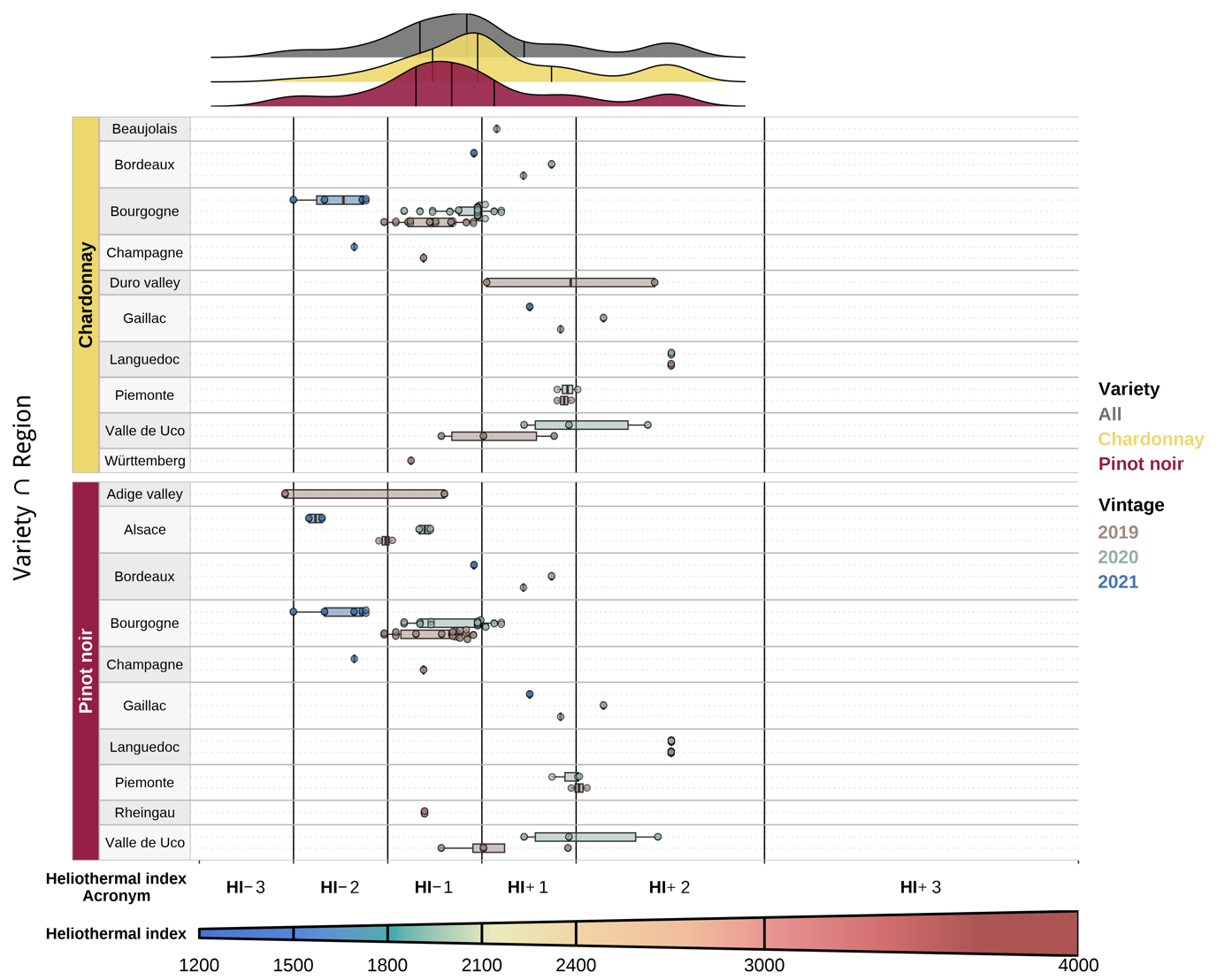


Fig. S4. Venn diagram representation of the variance partitioning of δ^13^C responses as explained by replicates of samples, region of origin, vintage and variety (i.e. grapevine cultivar).


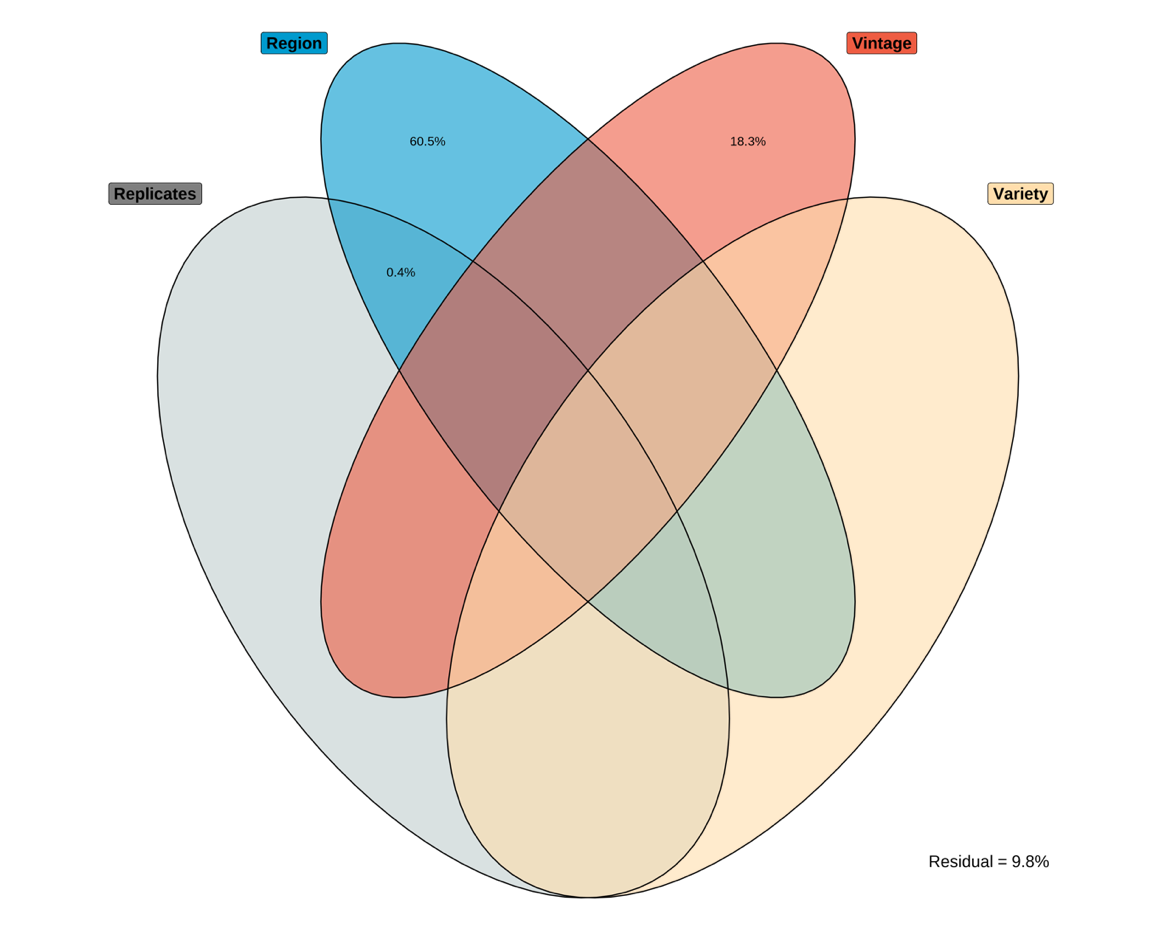


Fig. S5. Climagrams of hydric (Dryness) index and thermal (Huglin heliothermal) index. a, as depicted using TerraClimate gridded data (Abatzoglou et al., 2018) from observations (1971-2022) and projections according to +2°C and +4°C scenarios compared to preindustrial era over 20 years periods in each warming scenarios using the pattern scaling approach as depicted by (Qin et al., 2020). b.1-b.4, distinct climagrams for Bourgogne, Champagne, Valle de Uco and Bordeaux, with data from observations only, split in decades. The dryness index is a simplified monthly time step water balance model for grapevine adapted from Riou, C. (Riou, 1994) by Tonietto & Carbonneau, 2004. Huglin’s heliothermal index is a heat summation index proposed by Huglin (Huglin, 1978), modulated according to the average daylength during the grapevine growing season according to the latitude. Both indices are calculated from April to September for the Northern Hemisphere and from October to March for the southern hemisphere. For figure a, the amount of water added through irrigation is considered in the drynesss index, while for b.1-b.4, the dryness index is computed only based on precipitation.


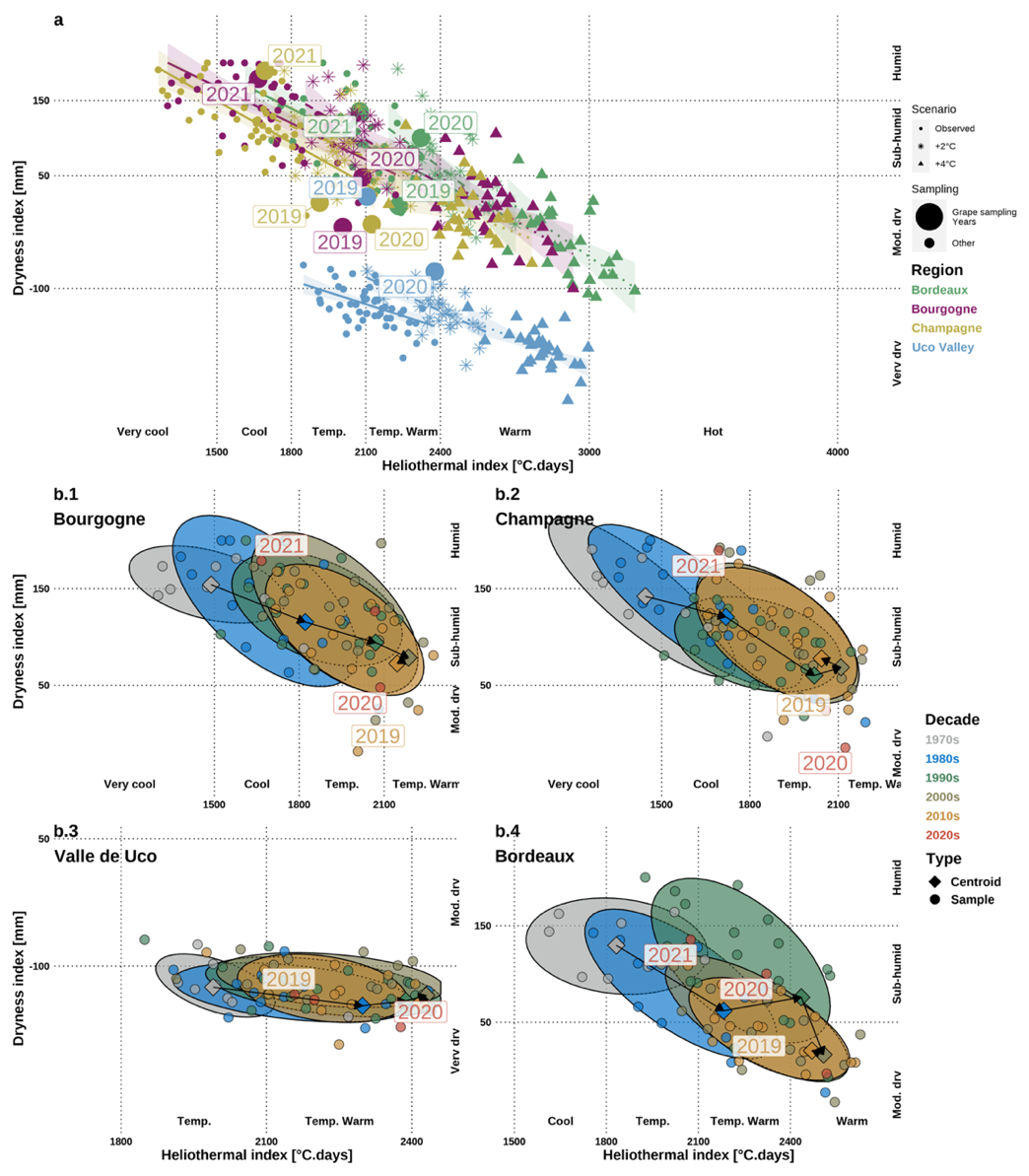


Fig. S6. Venn diagrams representations of the variance partitioning of the metabolomic data set (matrix of mass intensities obtained from FTIR-MS analyses) as explained by grapevine water status (δ^13^C), climate conditions (Dryness Index, DI, Huglin Index, HI (Huglin, 1978; Riou, 1994; Tonietto & Carbonneau, 2004) and variety (i.e. grapevine cultivar).


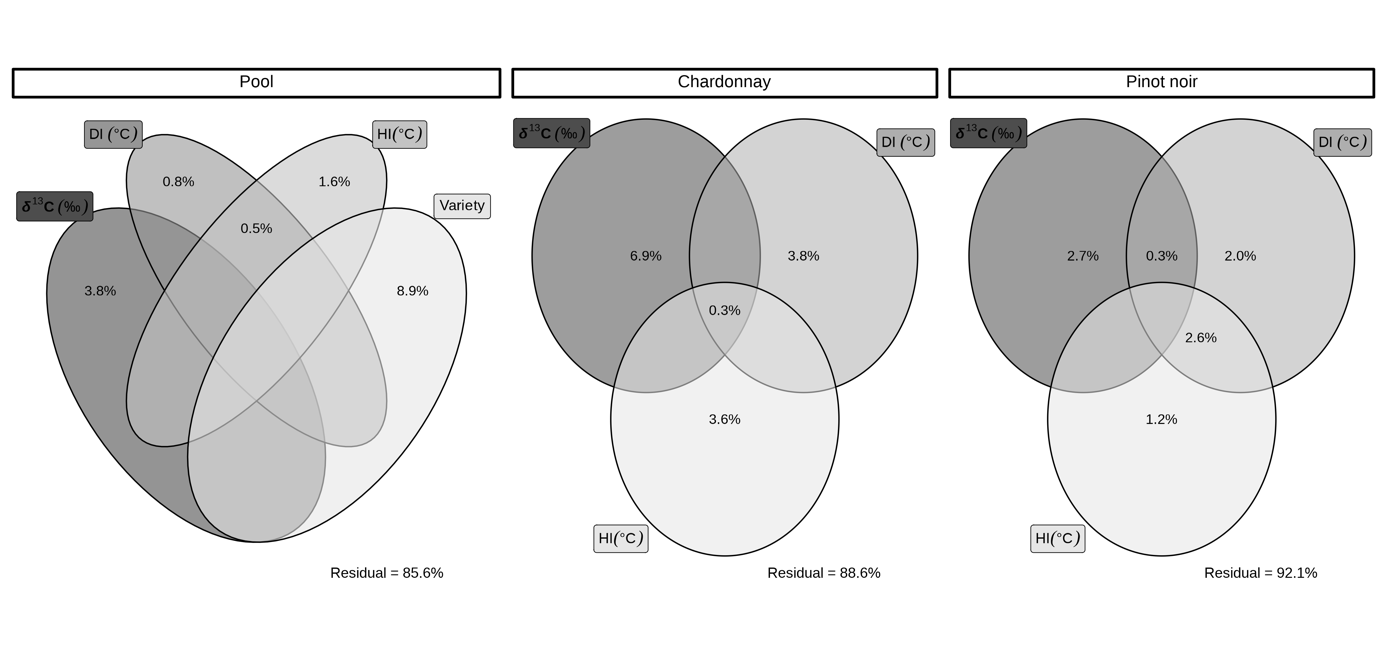


**Fig. S7.** **δ^13^C values, Dryness Index and Huglin Index distributions among all samples, by vintage.** For each vintage, data are presented as dots (given location) box plots and distributions. Red dots correspond to respective vintage means.
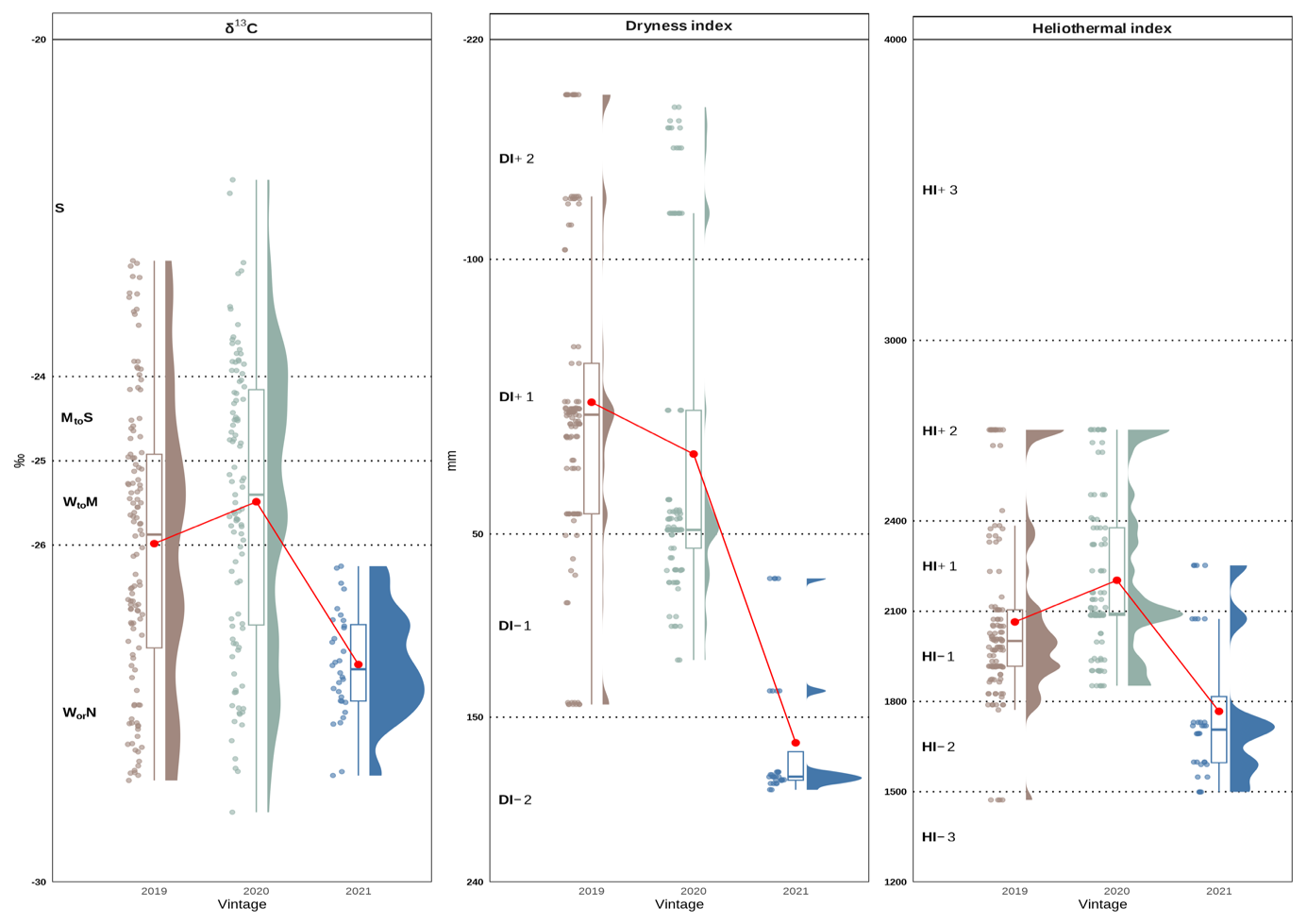


**Fig. S8. Metabolomics discrimination between Chardonnay and Pinot noir grape juices.** a, O-PLS-DA, and b**,** S-plot (Wiklund et al., 2008) where points are colored if O-PLS-DA VIP values are > 1.5, with color being associated to the grape variety with higher intensity (fold change tests). Grey dots correspond to VIP ≤ 1.5. c, distribution of identified VIPs in the different chemical classes: CHO (blue), CHNO (orange), CHOS (green), CHNOS (red). Results already presented in Nicolas et al., 2024, but with some modifications related to the number of samples analyzed, which was lower than that of the current study.


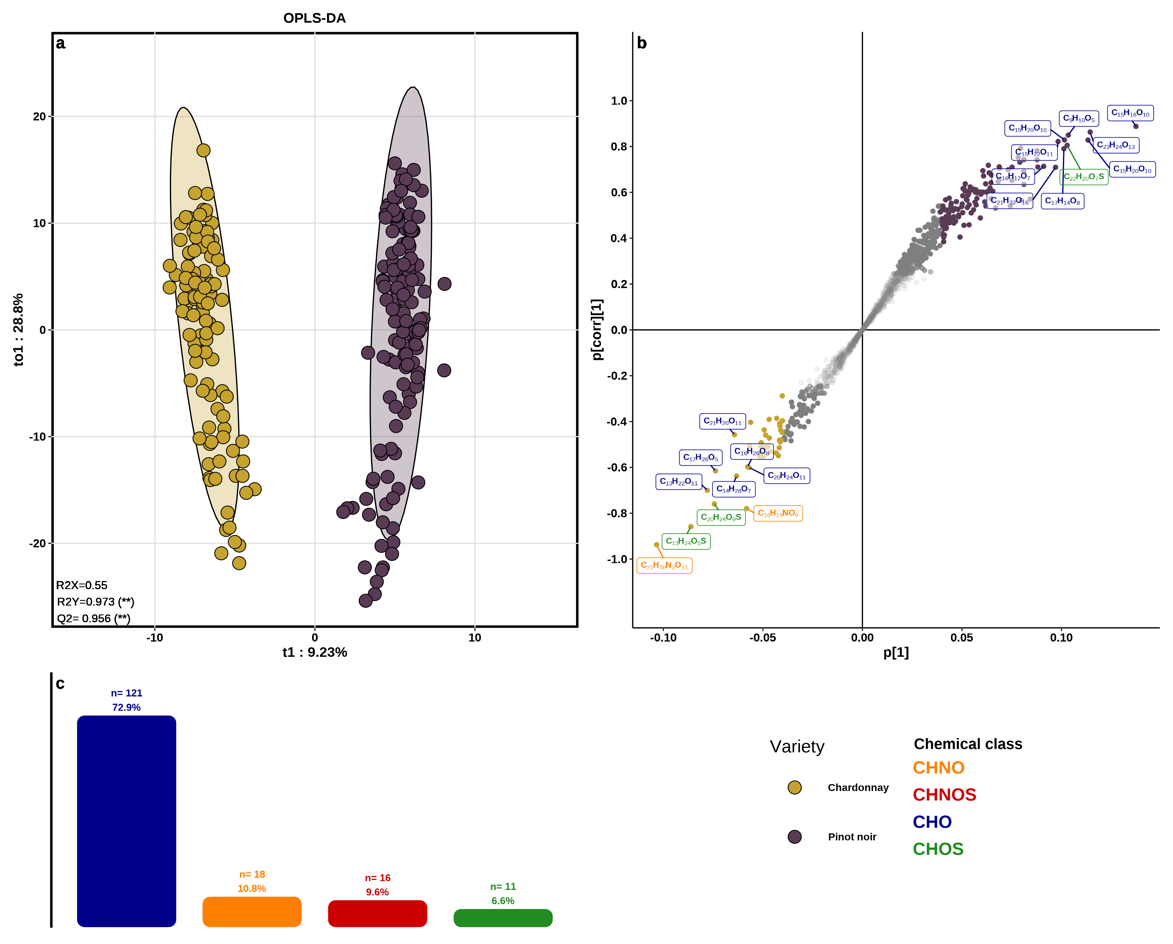


Fig. S9. S-plot representation of the δ^13^C-driven metabolomics datasets (a,c,e) and corresponding VIPs van Krevelen diagrams (b,d,f) for Chardonnay (a-b) and Pinot noir (c-d) and pooled datasets (e-f). Points are colored if O-PLS VIP values are > 1.5. The color code is associated to the chemical class (CHO (blue), CHNO (orange), CHOS (green), CHNOS (red)). For van Krevelen diagrams, bubbles marked with a circle/cross could be associated with low δ^13^C values (no water deficit).


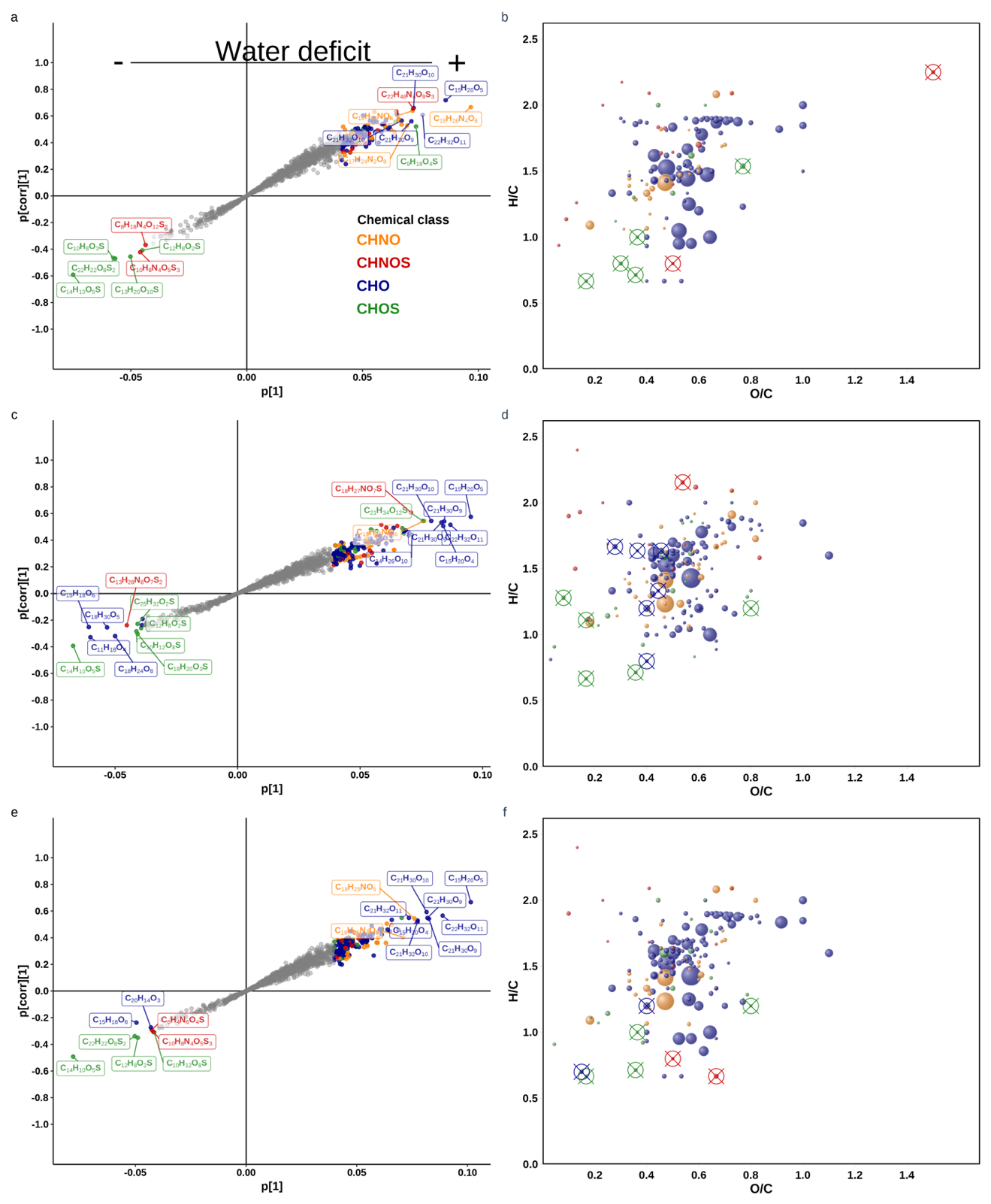


**Fig. S10.** **Counts of the number of elemental formulas, which appeared to be linked to δ^13^C values according to the grape variety.**  a. Volcano plot of masses, where only masses with significantly higher intensity in Chardonnay or in Pinot noir are colored (Mann-Whitney test, p-values correction: FDR). b. Histogram representation of these masses distributed in chemical classes (color code: CHO (blue), CHNO (orange), CHOS (green), CHNOS (red)), with indication of the annotation coverage using MetaCyc (black), PlantCyc (dark grey) and GrapeCyc (grey) databases (80,81).


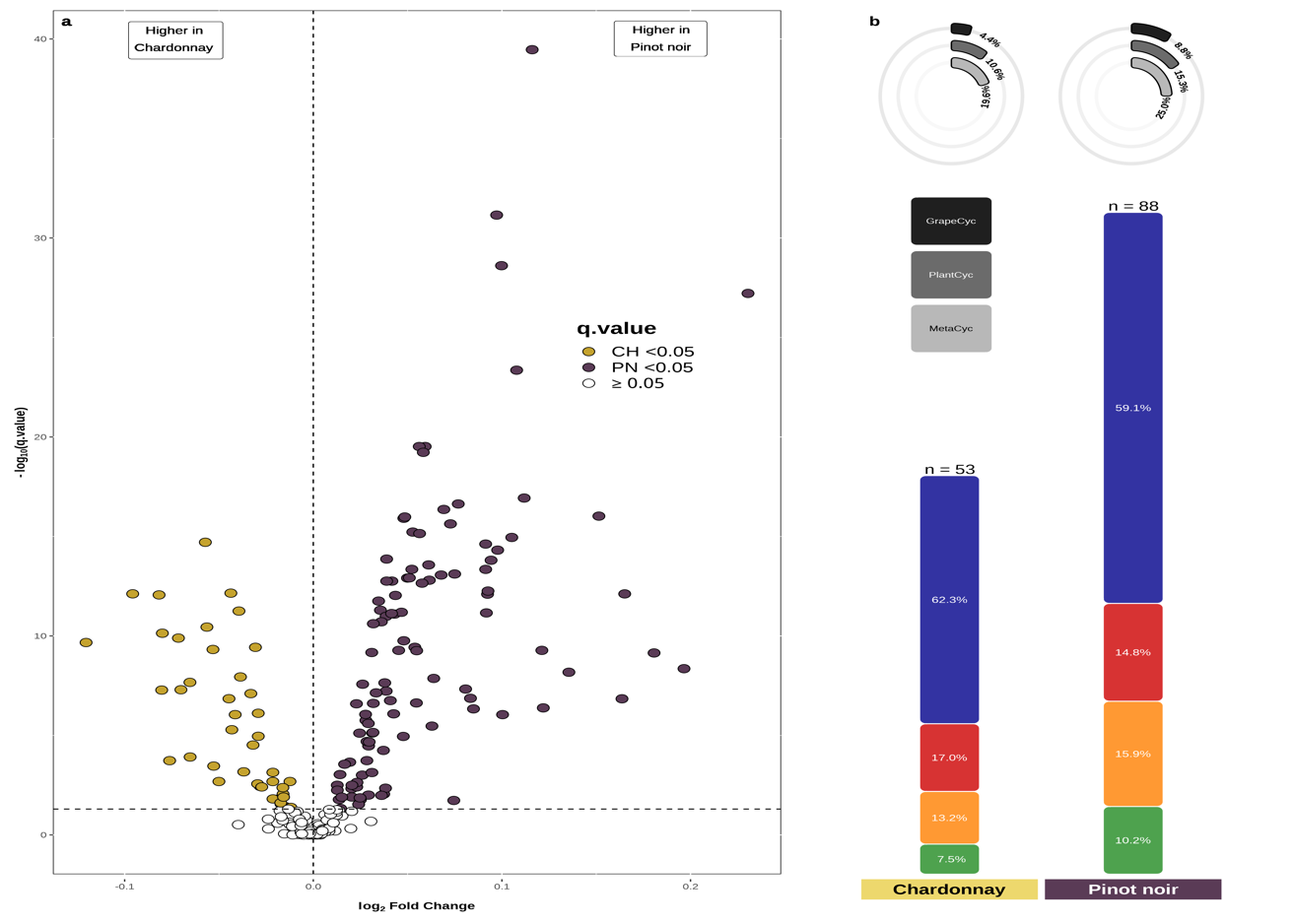


Fig. S11. Correlation networks among CHNOS elemental compositions computed for Chardonnay VIPs, identified using O-PLS analysis, with significant spearman correlation (|𝜌| >= 0.7, q.value (FDR) < 10^-5^). Count of computed mass differences with bio/chemical meaning in the VIPs network (upper left), and complete VIPs network (upper right). Each dot represents a VIP (mass peak transformed into elemental composition) and each line corresponds to a spearman correlation between two VIPs. Sub-networks corresponding to CHO (blue), CHOS (green), CHNO (orange) and CHNOS (red) elemental compositions are also represented. Bold lines in sub-networks indicate a spearman correlation with a further bio/chemical mass difference as listed in the upper left part (Breitling et al., 2006).


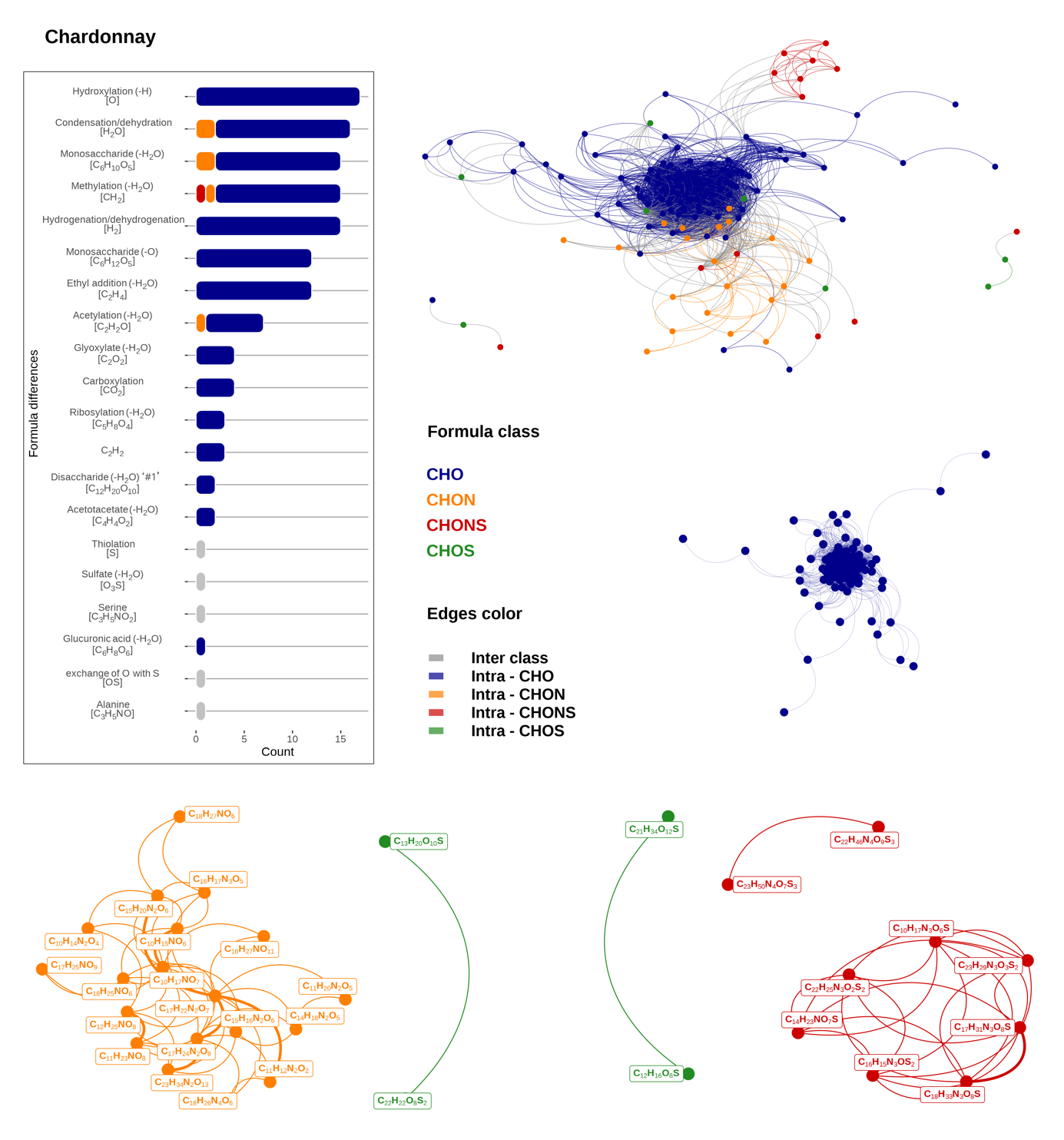


Fig. S12. Correlation networks among CHNOS elemental compositions computed for Pinot noir VIPs, identified using O-PLS analysis, with significant spearman correlation (|𝜌| >= 0.7, q.value (FDR) < 10^-5^). Count of computed mass differences with bio/chemical meaning in the VIPs network (upper left), and complete VIPs network (upper right). Each dot represents a VIP (mass peak transformed into elemental composition) and each line corresponds to a spearman correlation between two VIPs. Sub-networks corresponding to CHO (blue), CHOS (green), CHNO (orange) and CHNOS (red) elemental compositions are also represented. Bold lines in sub-networks indicate a spearman correlation with a further bio/chemical mass difference as listed in the upper left part (Breitling et al., 2006).


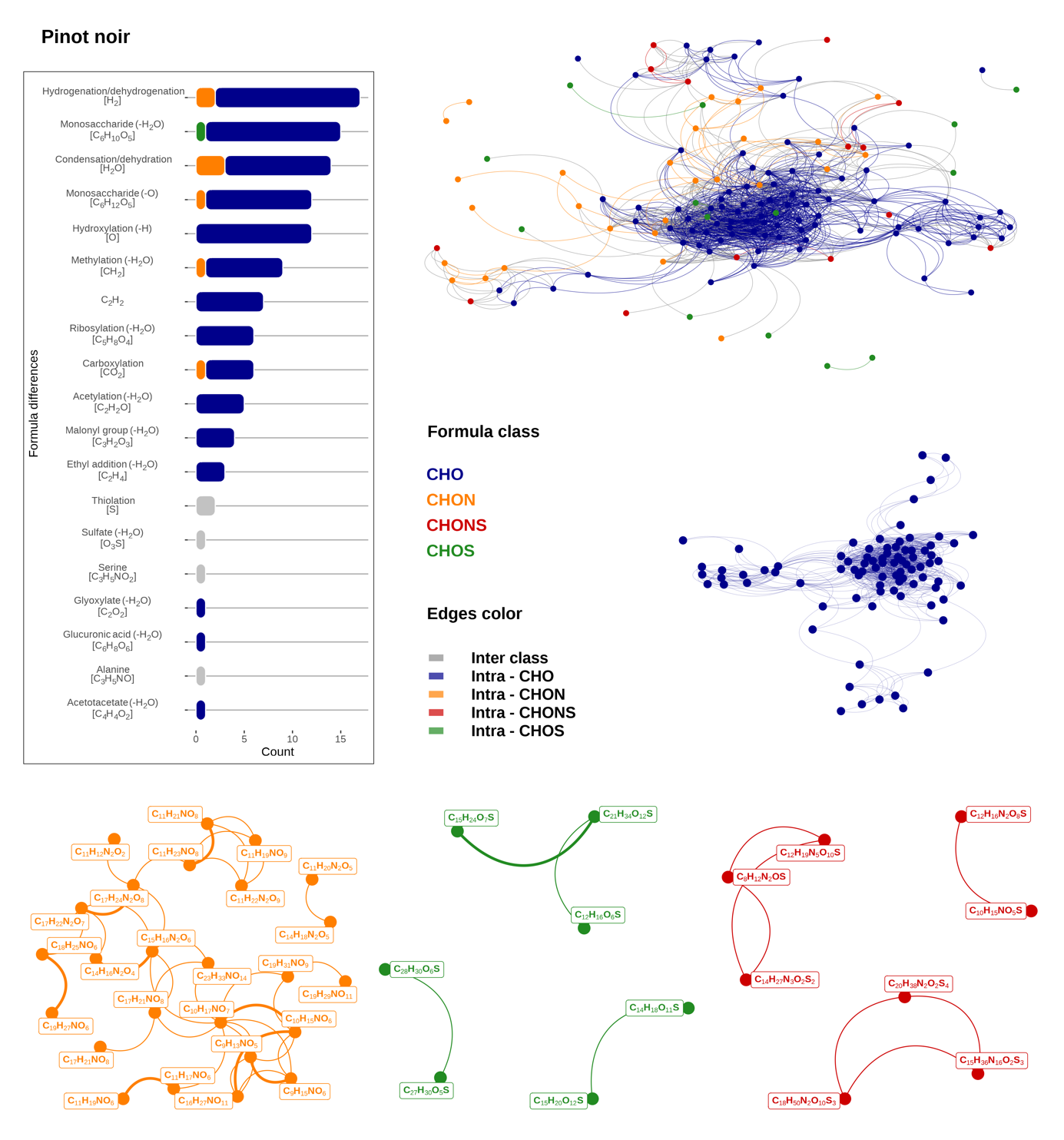


Fig. S13. Water status-correlated dynamics of all VIPs mass peak intensities, with assigned ion elemental compositions (color code: CHO (blue), CHNO (orange), CHOS (green), CHNOS (red)). Each dot corresponds to the Log representation of the associated mass peak intensity for a given sample (location x vintage x cultivar). Curves are produced from general additive models (GAM) fitting each cultivar (Chardonnay in yellow, Pinot noir in dark purple, Grey if the formula is not a VIP for a given cultivar). Stars on the right of the curves represent the GAM’s coefficient significance (* p <0.05, ** p <0.01, *** p <0.001). The line shaded areas around the curves correspond to the 95 % confidence interval. Finally, the upper part of each plot represents the marginal derivatives, which give information about the significance (plain line) of the relation along the water status gradient.


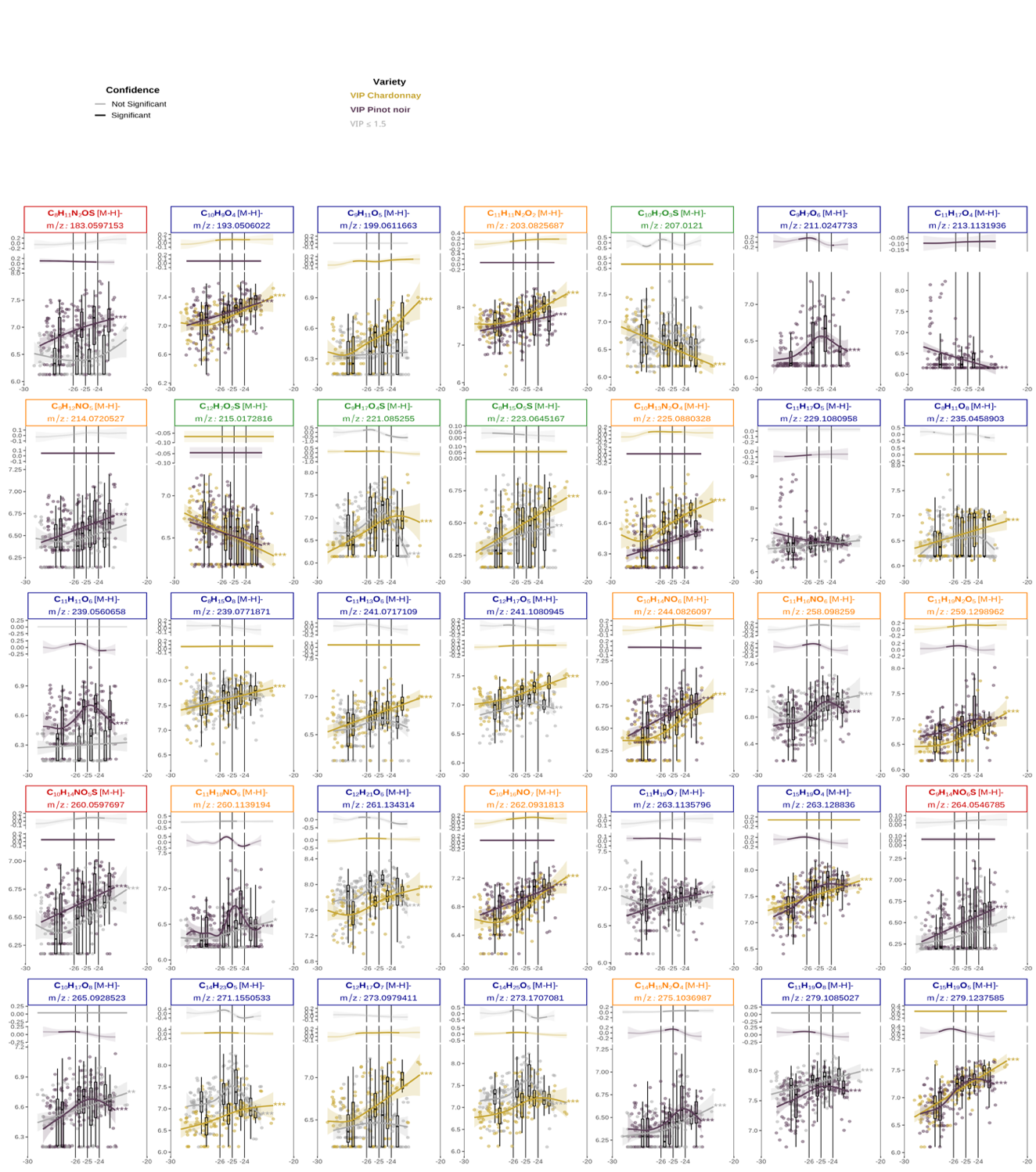


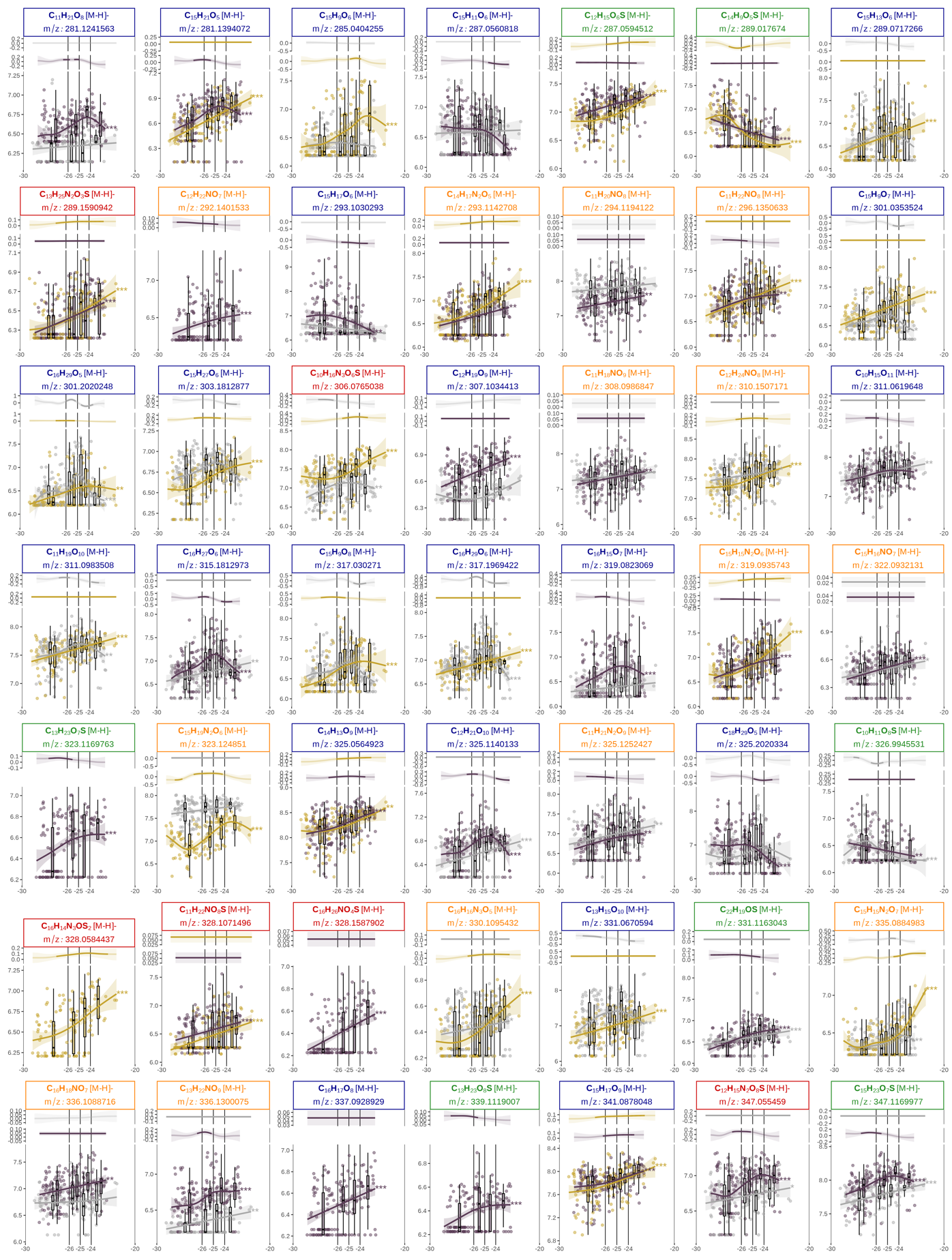


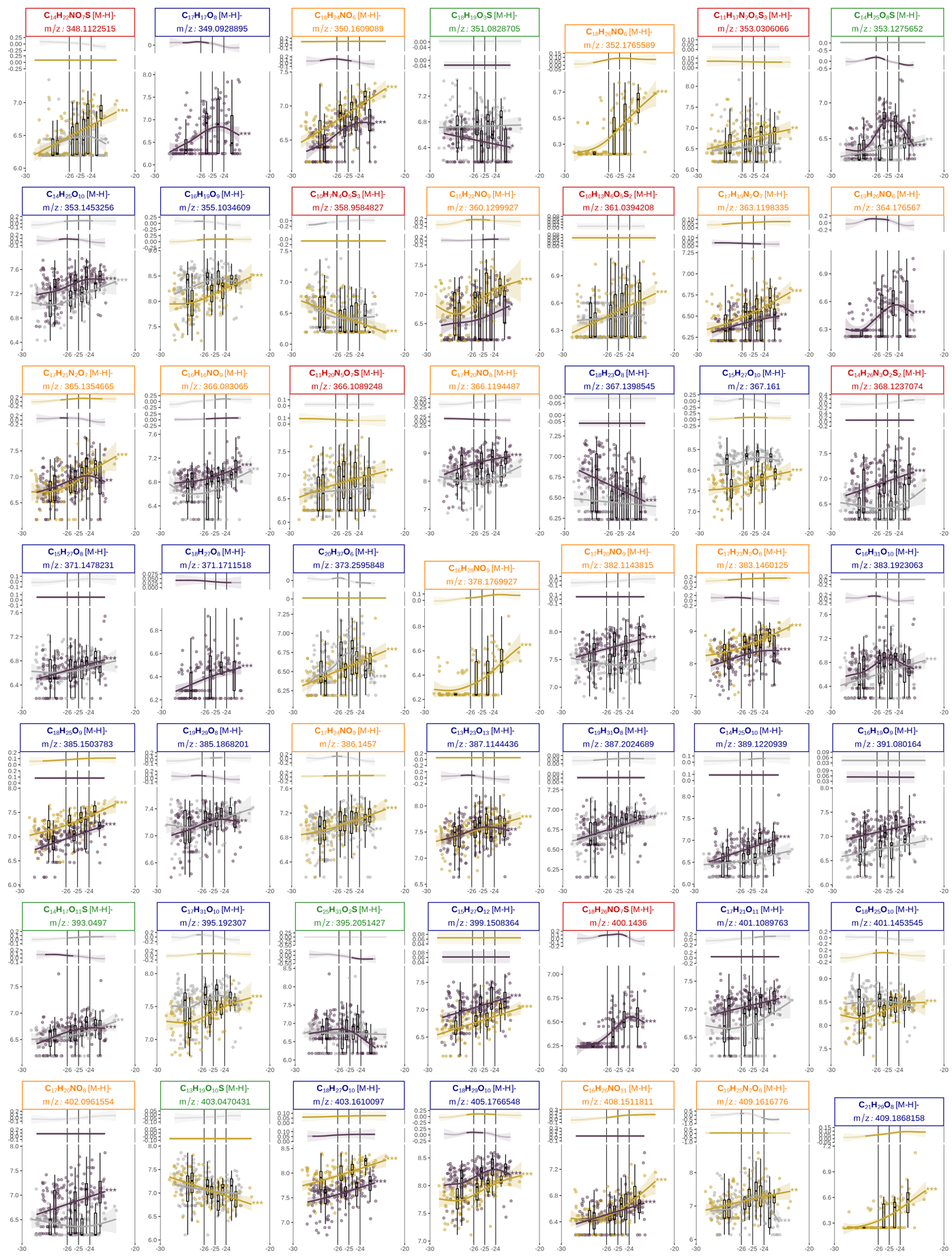


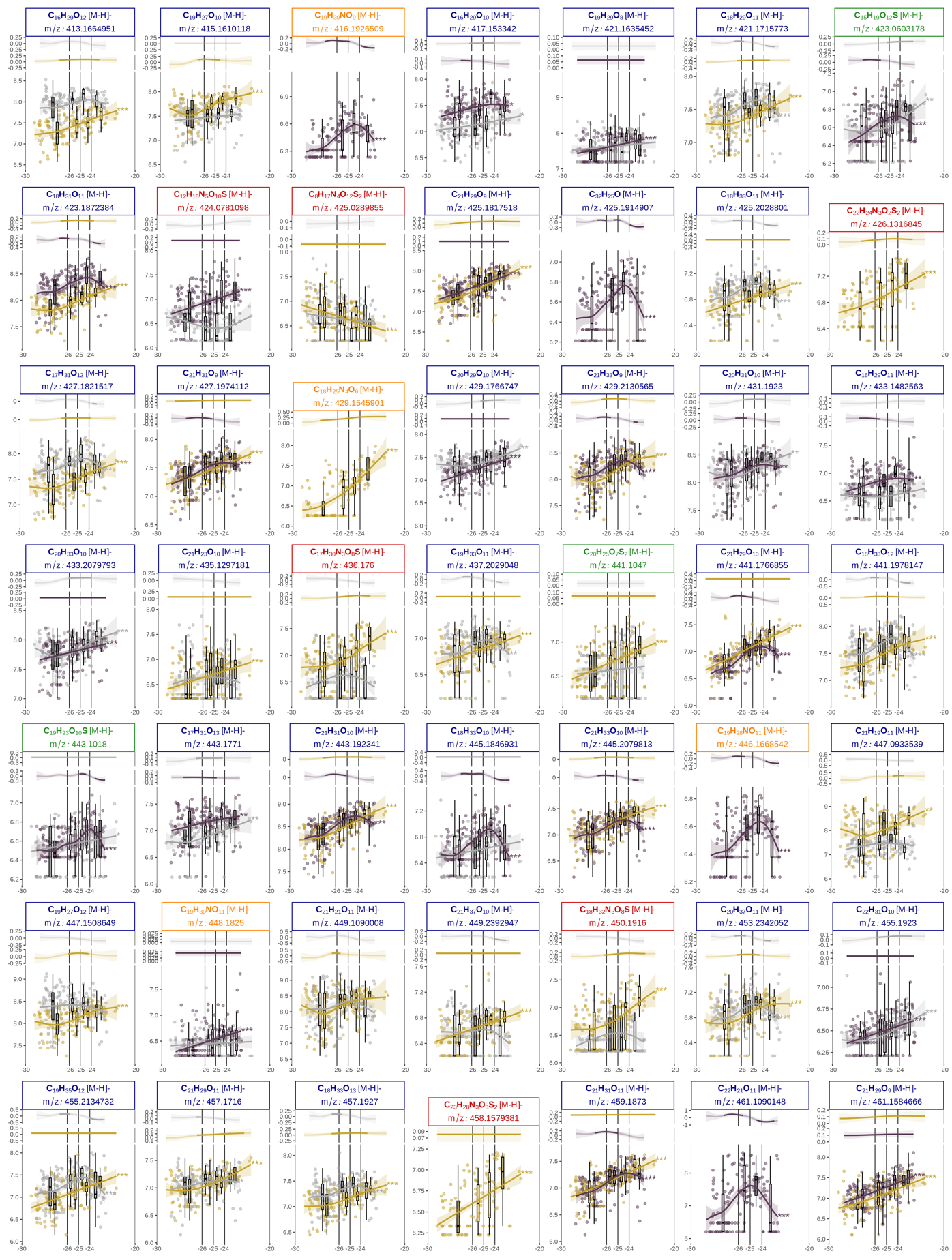


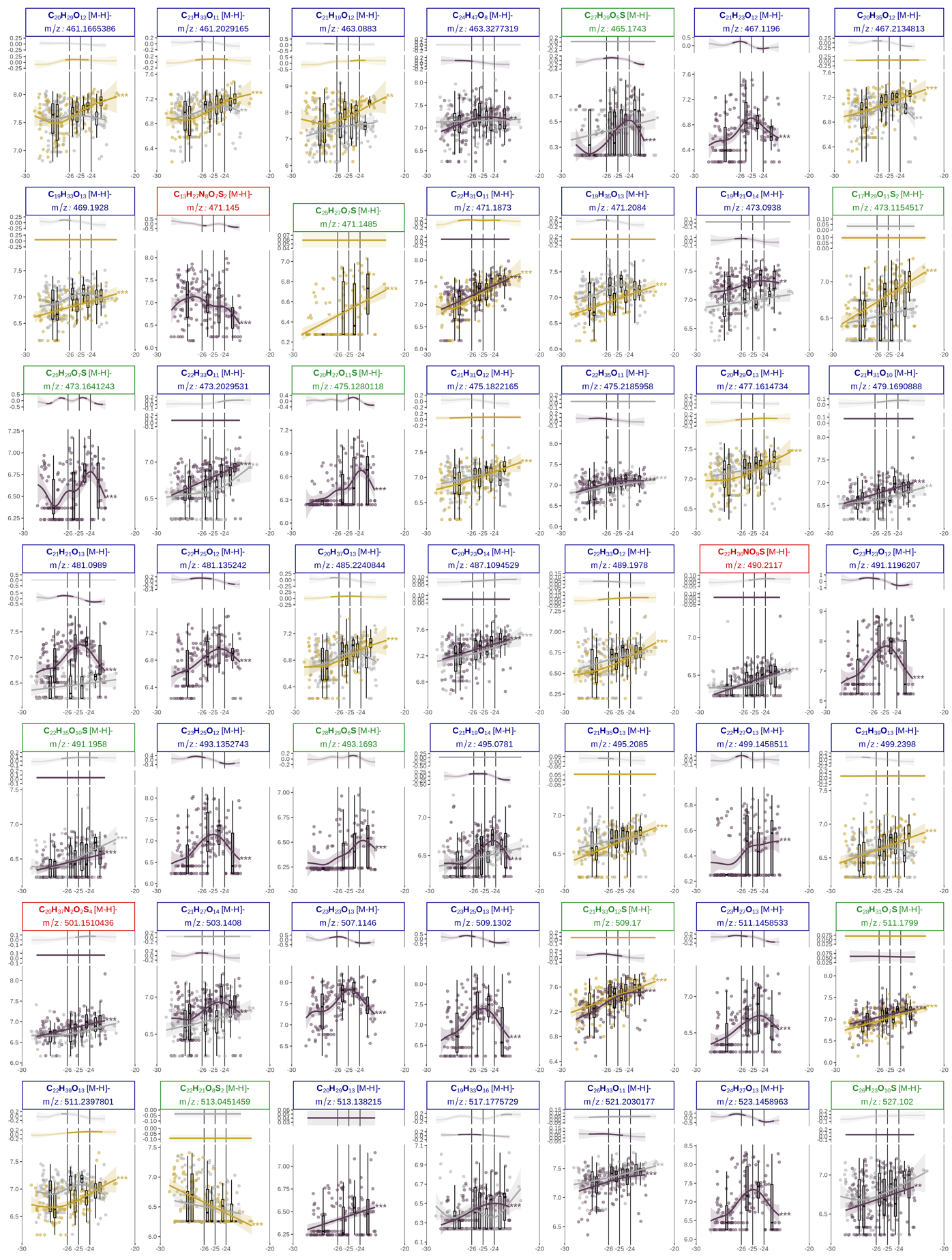


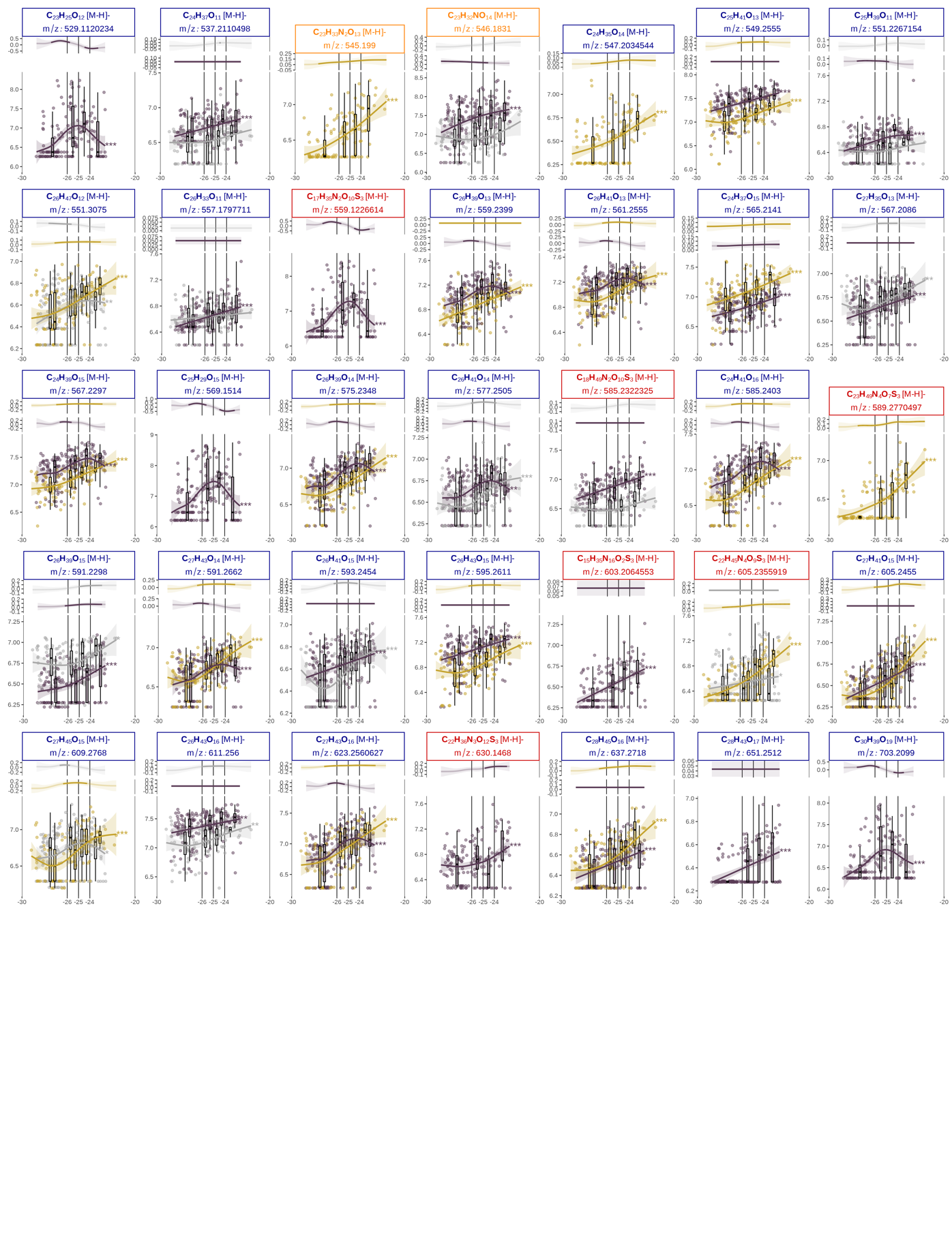


Fig. S14. δ^13^C class-based O-PLS score plots of pooled (PN & CH) metabolomics data (first column), along with van Krevelen diagrams representations of associated VIPs (second column), and corresponding frequencies of CHO (blue), CHOS (green), CHNO (orange) and CHNOS (red) elemental compositions (third column). Each row corresponds to an O-PLS performed on a data subset of δ^13^C values classified according to Santesteban et al., 2015 (colored scale at the top-left). van Krevelen diagrams and associated histograms represent VIPs >1.5 (mass peaks transformed into elemental formulas), whose relative intensity variation appeared either positively or negatively correlated with the increase of δ^13^C values.


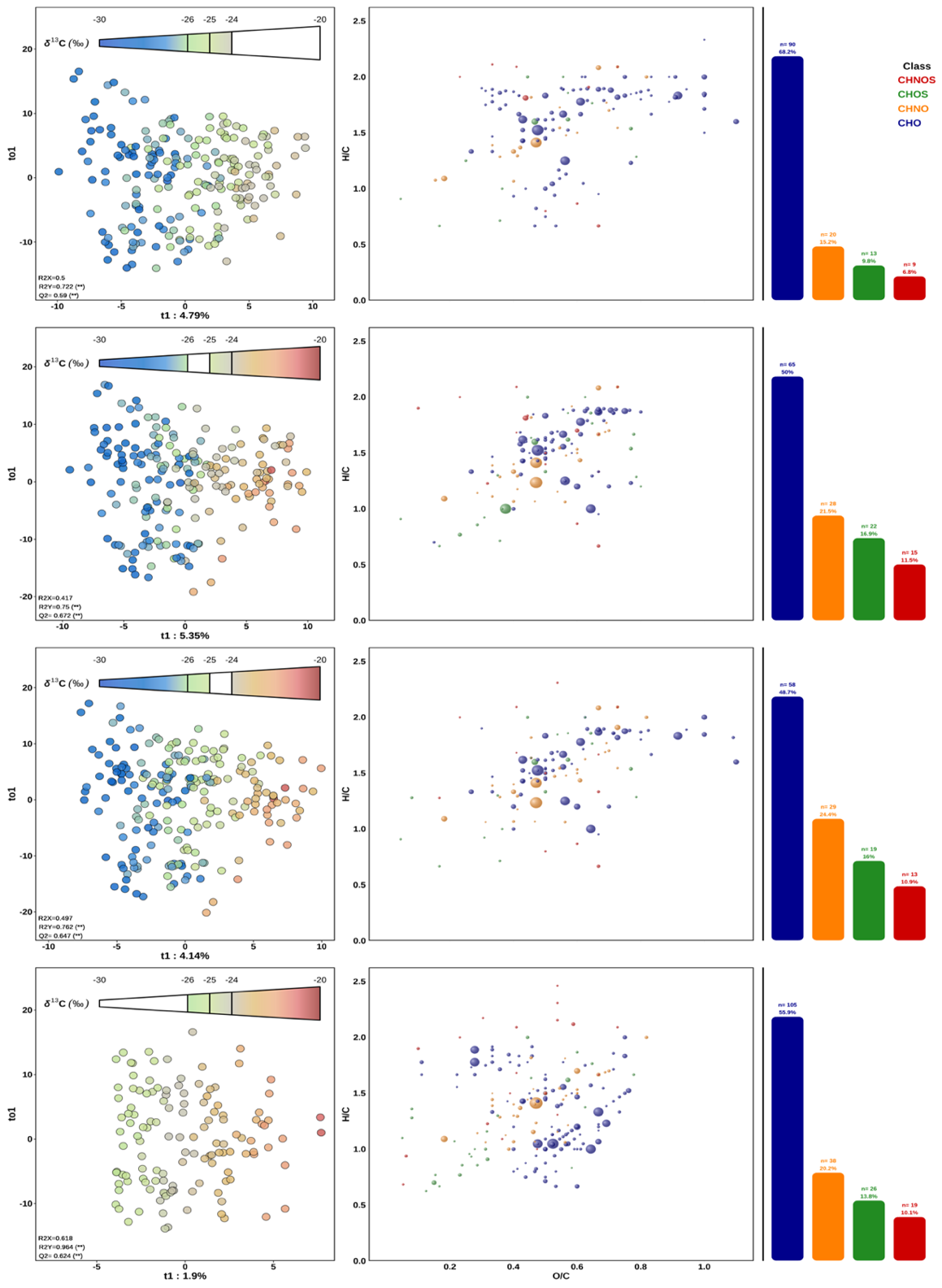


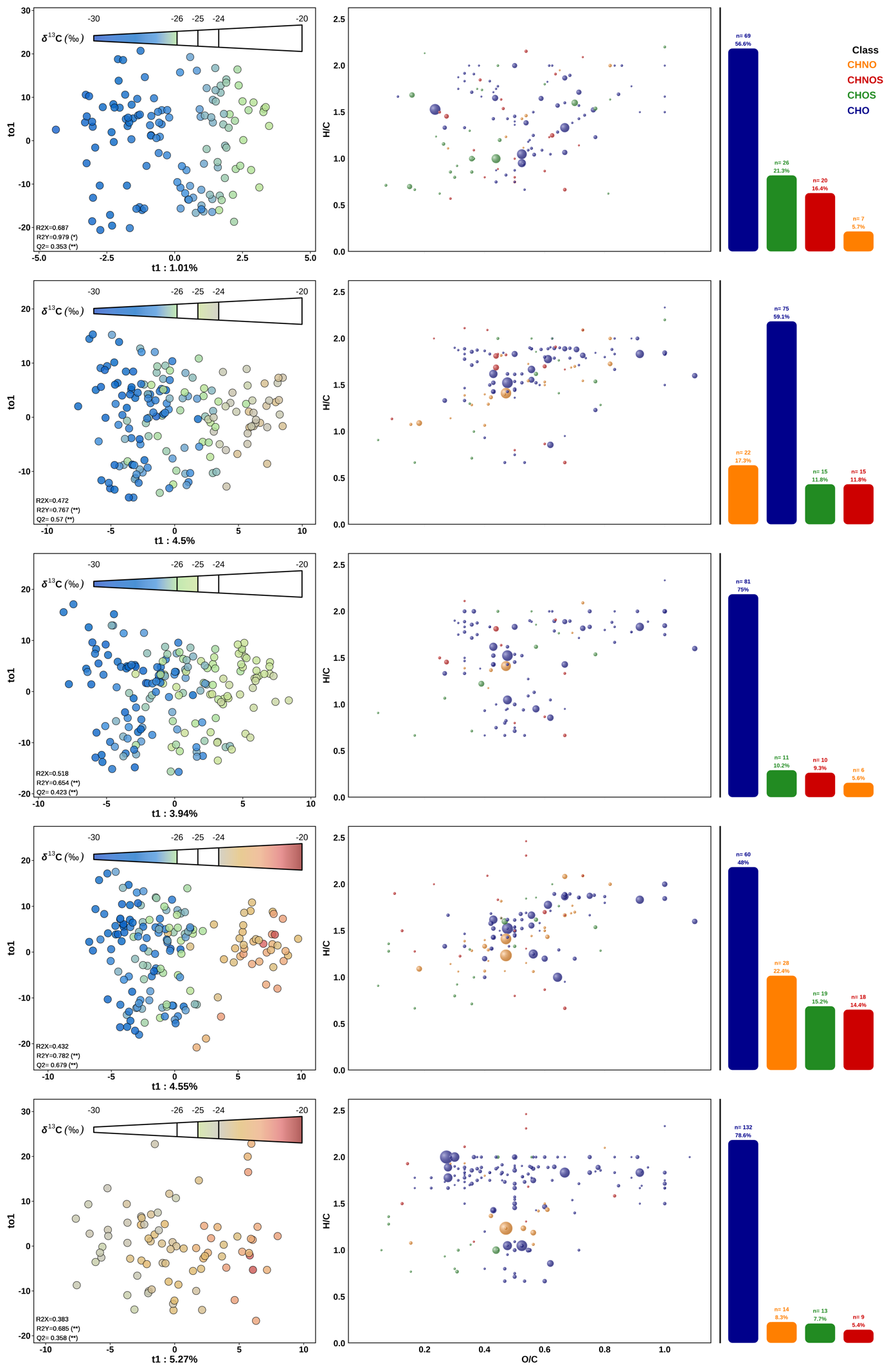


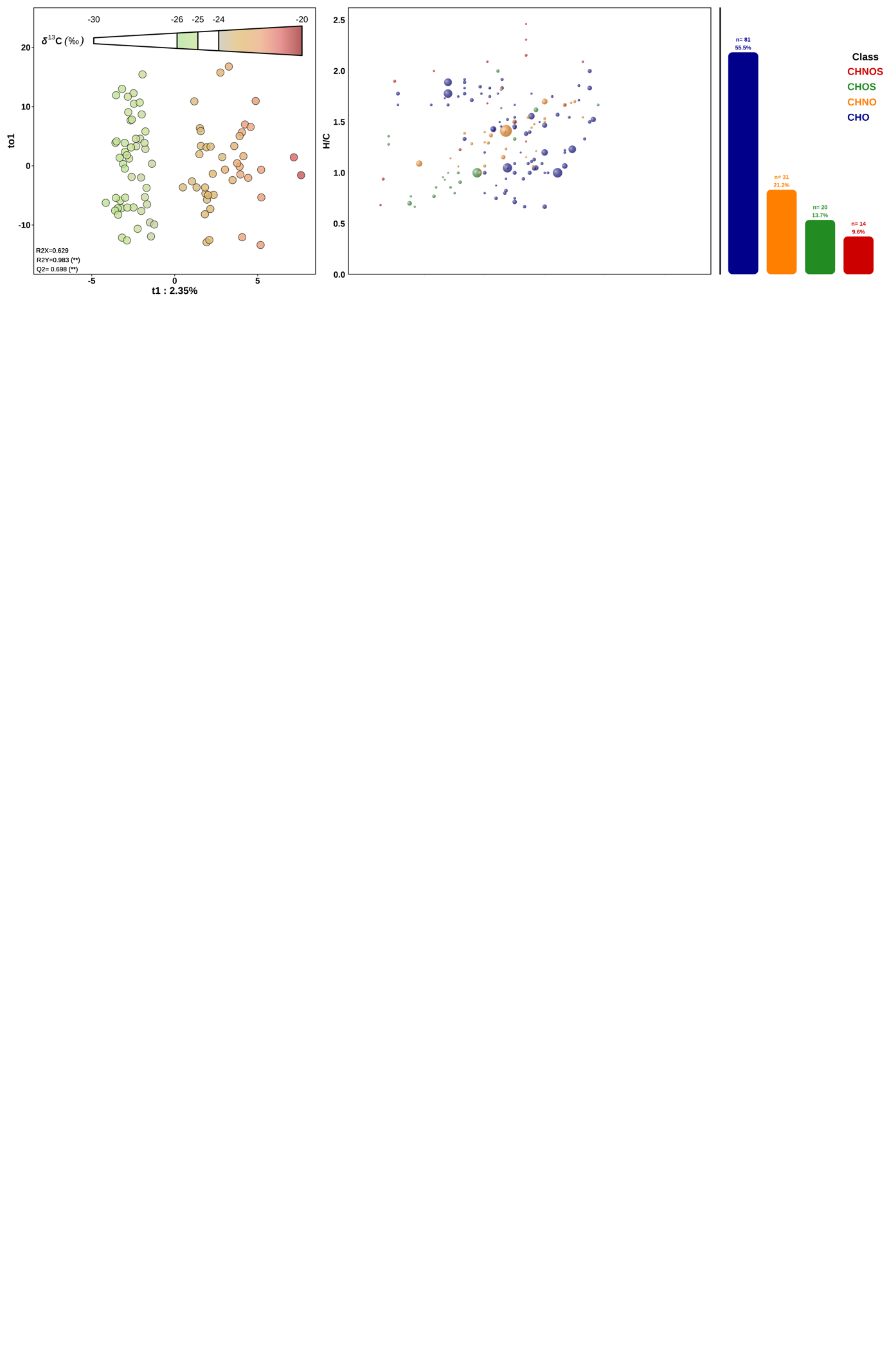


Fig. S15. δ^13^C class-based O-PLS score plots of Chardonnay metabolomics data (first column), along with van Krevelen diagrams representations of associated VIPs (second column), and corresponding frequencies of CHO (blue), CHOS (green), CHNO (orange) and CHNOS (red) elemental compositions (third column). Each row corresponds to an O-PLS performed on a data subset of δ^13^C values classified according to Santesteban et al., 2015 (colored scale at the top-left). van Krevelen diagrams and associated histograms represent VIPs >1.5 (mass peaks transformed into elemental formulas), whose relative intensity variation appeared either positively or negatively correlated with the increase of δ^13^C values.


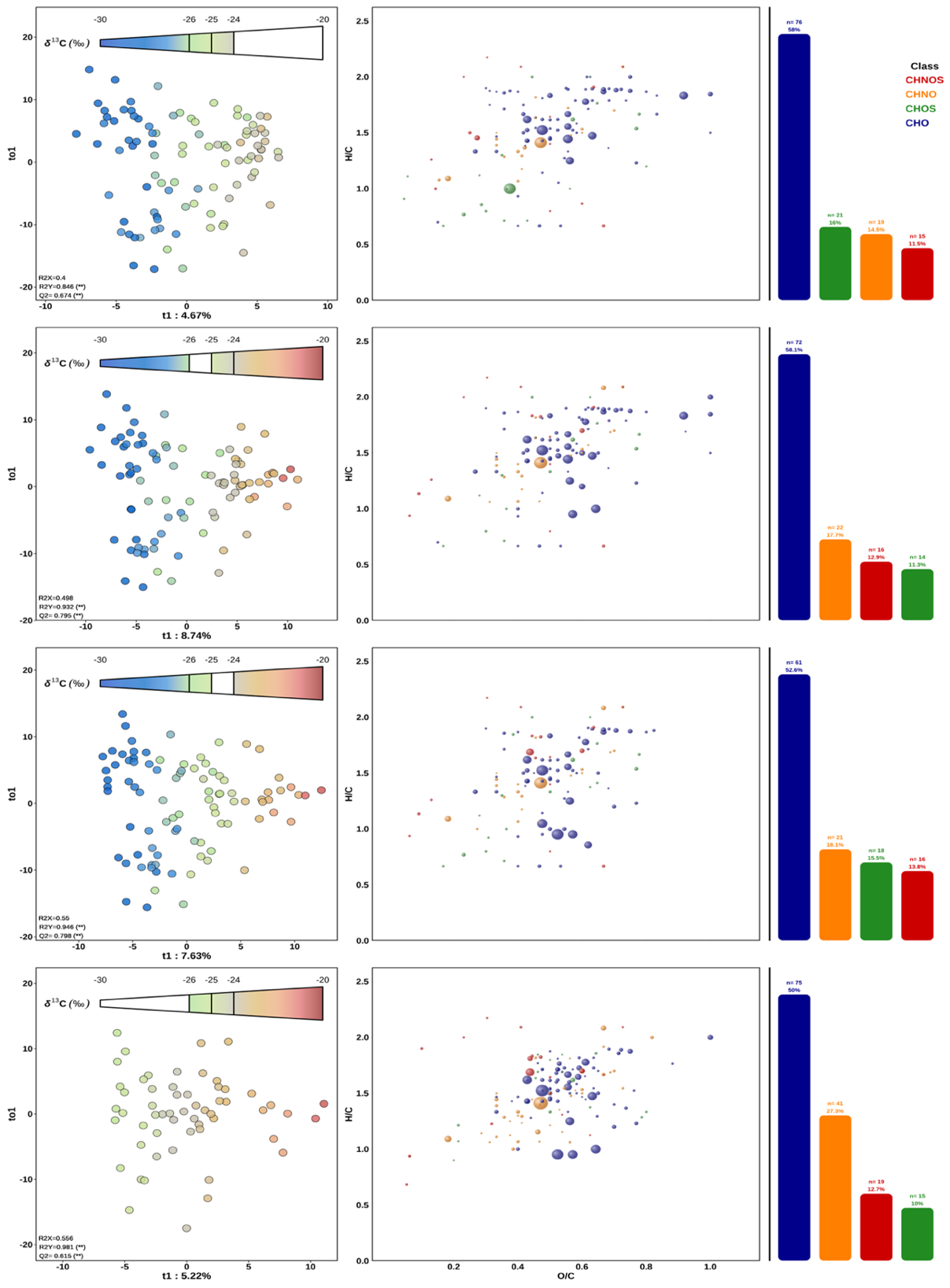


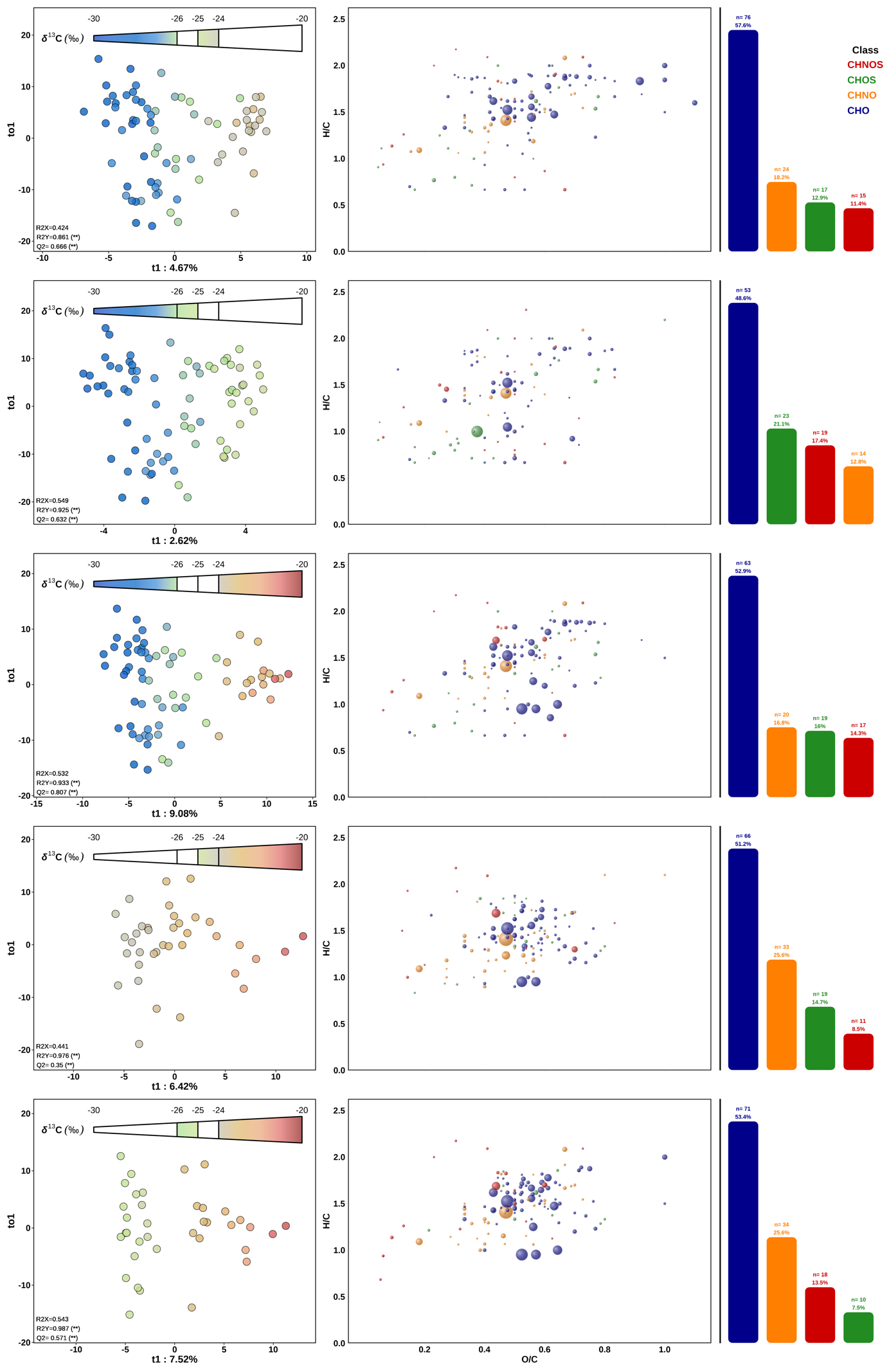


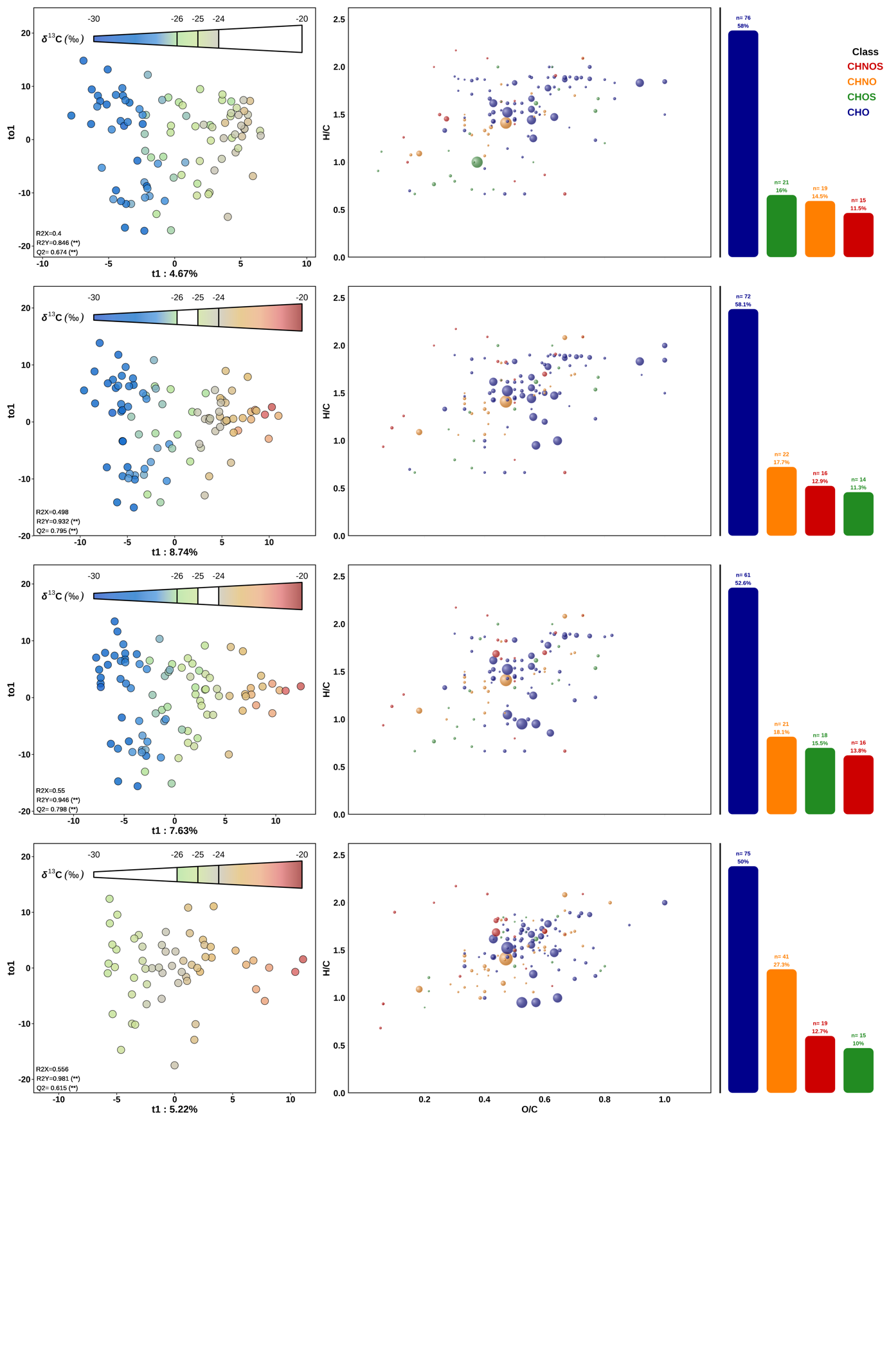


Fig. S16. δ^13^C class-based O-PLS score plots of Pinot noir metabolomics data (first column), along with van Krevelen diagrams representations of associated VIPs (second column), and corresponding frequencies of CHO (blue), CHOS (green), CHNO (orange) and CHNOS (red) elemental compositions (third column). Each row corresponds to an O-PLS performed on a data subset of δ^13^C values classified according to (Santesteban et al., 2015) (colored scale at the top-left). van Krevelen diagrams and associated histograms represent VIPs >1.5 (mass peaks transformed into elemental formulas), whose relative intensity variation appeared either positively or negatively correlated with the increase of δ^13^C values.


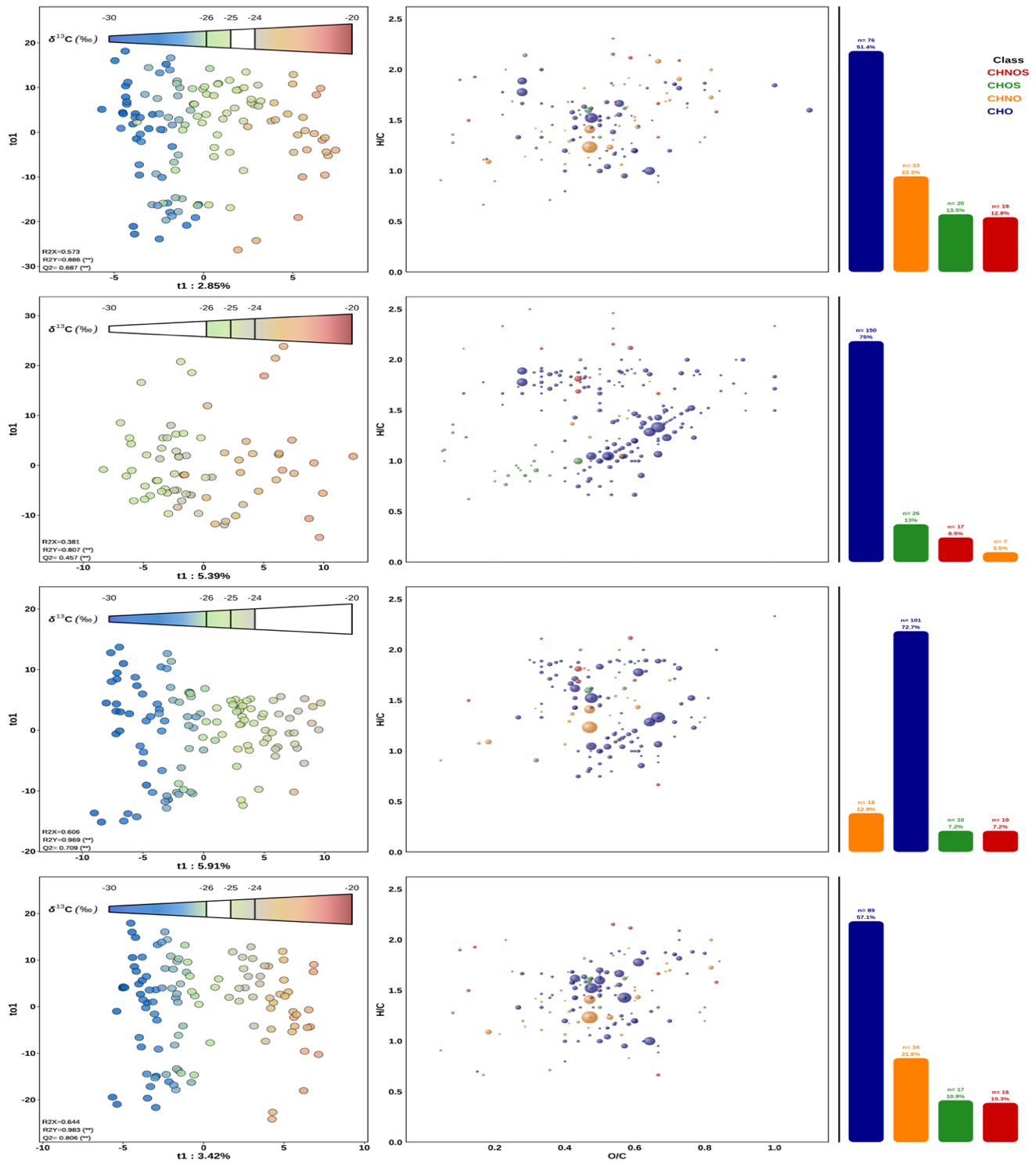


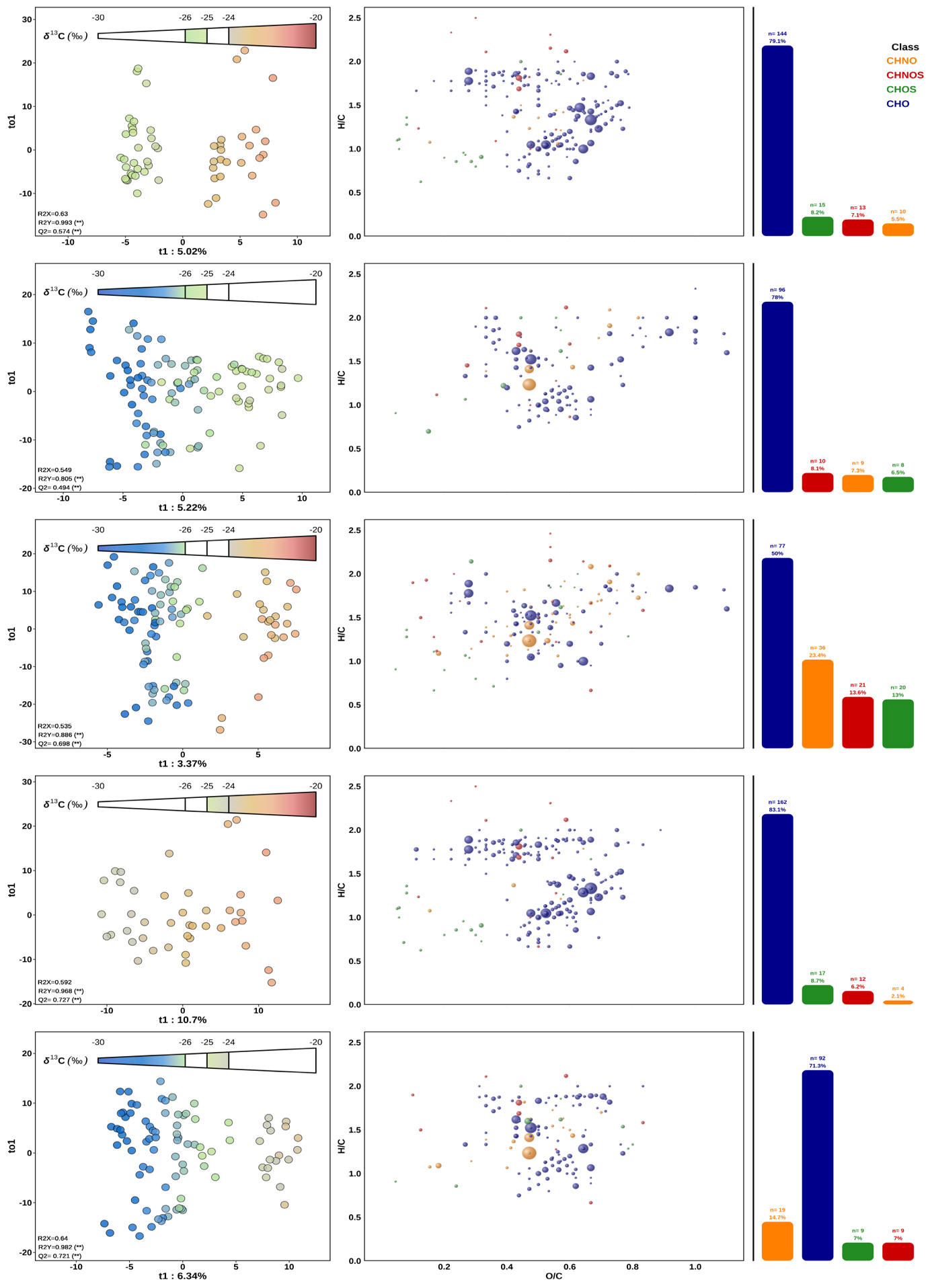


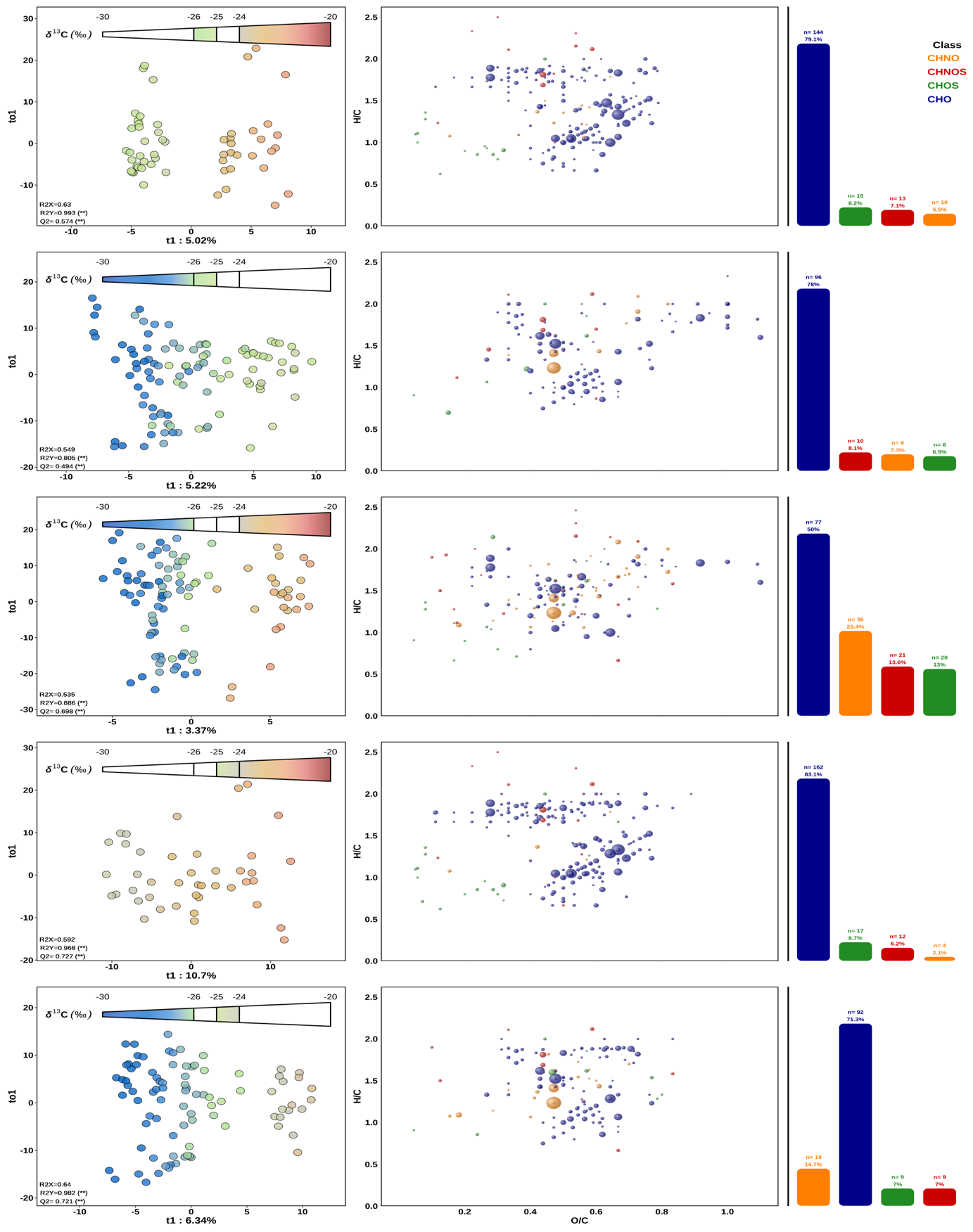


Supplementary References

Abatzoglou, J. T., Dobrowski, S. Z., Parks, S. A., & Hegewisch, K. C. (2018). TerraClimate, a high-resolution global dataset of monthly climate and climatic water balance from 1958-2015. *Scientific Data*, *5*, 1–12. https://doi.org/10.1038/sdata.2017.191

Breitling, R., Ritchie, S., Goodenowe, D., Stewart, M. L., & Barrett, M. P. (2006). Ab initio prediction of metabolic networks using Fourier transform mass spectrometry data. *Metabolomics*, *2*(3), 155–164. https://doi.org/10.1007/s11306-006-0029-z

Huglin, M. (1978). *Nouveau mode d’évaluation des possibilités héliothermiques d’un milieu viticole*.

Nicolas, S., Bois, B., Billet, K., Romanet, R., Bahut, F., Uhl, J., Schmitt-Kopplin, P., & Gougeon, R. D. (2024). High-Resolution Mass Spectrometry-Based Metabolomics for Increased Grape Juice Metabolite Coverage. *Foods*, *13*(1). https://doi.org/10.3390/foods13010054

Qin, Y., Abatzoglou, J. T., Siebert, S., Huning, L. S., AghaKouchak, A., Mankin, J. S., Hong, C., Tong, D., Davis, S. J., & Mueller, N. D. (2020). Agricultural risks from changing snowmelt. *Nature Climate Change*, *10*(5), 459–465. https://doi.org/10.1038/s41558-020-0746-8

Riou, C. (1994). Le déterminisme climatique de la maturation du raisin : application au zonage de la teneur en sucre dans la Communauté Européenne. *Agriculture Series. Office Des Publications Officielles Des Communautés Européennes, Luxembourg (FRA), 322 Pp.*

Santesteban, L. G., Miranda, C., Barbarin, I., & Royo, J. B. (2015). Application of the measurement of the natural abundance of stable isotopes in viticulture: A review. *Australian Journal of Grape and Wine Research*, *21*(2), 157–167. https://doi.org/10.1111/ajgw.12124

Tonietto, J., & Carbonneau, A. (2004). *A multicriteria climatic classification system for grape-growing regions worldwide*. *124*, 81–97. https://doi.org/10.1016/j.agrformet.2003.06.001

Wiklund, S., Johansson, E., Sjöström, L., Mellerowicz, E. J., Edlund, U., Shockcor, J. P., Gottfries, J., Moritz, T., & Trygg, J. (2008). Visualization of GC/TOF-MS-Based Metabolomics Data for Identification of Biochemically Interesting Compounds Using OPLS Class Models. *Analytical Chemistry*, *80*(1), 115–122. https://doi.org/10.1021/ac0713510
